# Design, synthesis and molecular docking of new fused 1*H*-pyrroles, pyrrolo[3,2-*d*]pyrimidines and pyrrolo[3,2-*e*][1, 4]diazepine derivatives as potent EGFR/CDK2 inhibitors

**DOI:** 10.1080/14756366.2022.2096019

**Published:** 2022-07-08

**Authors:** Amany Belal, Nagwa M. Abdel Gawad, Ahmed B. M. Mehany, Mohammed A. S. Abourehab, Hazem Elkady, Ahmed A. Al‐Karmalawy, Ahmed S. Ismael

**Affiliations:** a Medicinal Chemistry Department, Faculty of Pharmacy, Beni-Suef University, Beni-Suef, Egypt; b Department of Pharmaceutical Chemistry, College of Pharmacy, Taif University, Taif, Saudi Arabia; c Medicinal Chemistry Department, Faculty of Pharmacy, Cairo University, Giza, Egypt; d Department of Zoology, Faculty of Science, Al-Azhar University, Nasr City, Egypt; e Department of Pharmaceutics, Faculty of Pharmacy, Umm Al-Qura University, Makkah, Saudi Arabia; f Department of Pharmaceutics and Industrial Pharmacy, College of Pharmacy, Minia University, Minia, Egypt; g Pharmaceutical Medicinal Chemistry & Drug Design Department, Faculty of Pharmacy (Boys), Al-Azhar University, Cairo, Egypt; h Department of Pharmaceutical Medicinal Chemistry, Faculty of Pharmacy, Horus University‐ Egypt, New Damietta, Egypt

**Keywords:** Anticancer, 1*H*-pyrrole, pyrrolo[3,2-*d*]pyrimidine, pyrrolo[3,2-*e*][1, 4]diazepine, EGFR inhibitor, CDK2 inhibitor

## Abstract

A new series of 1*H*-pyrrole (**6a–c**, **8a–c**), pyrrolo[3,2-*d*]pyrimidines (**9a–c**) and pyrrolo[3,2-*e*][1, 4]diazepines (**11a–c**) were designed and synthesised. These compounds were designed to have the essential pharmacophoric features of EGFR Inhibitors, they have shown anticancer activities against HCT116, MCF-7 and Hep3B cancer cells with IC_50_ values ranging from 0.009 to 2.195 µM. IC_50_ value of doxorubicin is 0.008 µM, compounds **9a** and **9c** showed IC_50_ values of 0.011 and 0.009 µM respectively against HCT-116 cells. Compound **8b** exerted broad-spectrum activity against all tested cell lines with an IC_50_ value less than 0.05 µM. Compound **8b** was evaluated against a panel of kinases. This compound potently inhibited CDK2/Cyclin A1, DYRK3 and GSK3 alpha kinases with 10–23% compared to imatinib (1–10%). It has also arrested the cell cycle of MCF-7 cells at the S phase. Its antiproliferative activity was further augmented by molecular docking into the active sites of EGFR and CDK2 cyclin A1.

## 1. Introduction

Cancer is clearly associated with an increased incidence and rates of mortality, in addition to its devastating social and economic effects^1^^,^^2^. Cancer is a generic term for a large group of diseases that can affect any part of the human body^2^^,^^3^. The complexity of cancer pathologies manifests in oncogenic mutations, severe and occasionally fatal drug side effects, multi-drug resistance and activation of compensatory pathways^4–6^. Thus, there is an urgent need to develop more efficient anticancer candidates, relying on various biological and molecular aspects of neoplastic transformation. Accordingly, many efforts were done to reach promising anticancer candidates^1^^,^^7–10^.

Protein kinases constitute one of the largest and most functionally diverse gene families that control a diverse set of cellular processes. Thus, they perform a major role in the proliferation, metastasis and survival of human tumour cells^11^. Tyrosine kinase is the most important protein kinases sub-family. This family comprises epidermal growth factor receptor (EGFR)^12^, vascular endothelial growth factor receptor (VEGFR)^13^, fibroblast growth factor receptor (FGFR)^14^ and cyclin-dependent kinases (CDK)^15^. Three of the protein kinases have been crystallised in active conformations (cAPK, PhK, and CK1), one in a partially active conformation (CDK2 in complex with cyclin A), and five in inactive conformations (CDK2, MAPK, IRK, twitchin kinase, and CaMKI). The structures of the kinases in the active conformation all showed equivalent positions for essential catalytic site residues Lys-72, Asp-166, and Asp-184 in cAPK. Their positions and the correct orientation of the Mg^2+^/ATP and the protein substrate appear crucial for catalysis and are dependent upon the tertiary structure of both lobes and the correct relative orientation of the lobes. In the partially active CDK2–cyclin A complex, the charge grouping is compensated by glutamate, Glu-162, (two residues removed from the phosphorylatable threonine Thr-160) and by interactions of arginyl residues with the main chain carbonyl groups from cyclin A. In PhK, there is only one basic group, the arginine adjacent to the catalytic base, and charge compensation can be satisfactorily accomplished by a carboxylate group^16^.

The epidermal growth factor receptor (EGFR) is a transmembrane protein receptor endowed with tyrosine kinase activity, occupying an important position in cancer progression and tumour cell biology. EGFR plays a role in cellular phenotyping and provides tumour cells with significant growth^17^. Elevated levels of EGFR and its associated ligands (EGF and transforming growth factor (TGF)) have been found to be a common feature of a variety of cancer types. In many cases, abnormal EGFR activation appears to be a key element in carcinogenesis and a major driving force for cancer growth^18^. Increased EGFR expression is thus expected to be a powerful prognostic factor in a variety of tumour forms and blocking its cellular functions looks to have significant therapeutic effects^19^. As a result, EGFR is being regarded as a viable target for the development of novel anticancer drugs^20–23^.

The cyclin-dependent kinases (CDKs) are a group of enzymes involved in cell cycle progression and cellular proliferation^24^. They work by phosphorylating critical serine and threonine residues in host proteins, which then can activate them^25^^,^^26^. It is commonly believed that inhibiting CDKs could help the limitation of the uncontrolled cellular proliferation seen in some malignancies^27^. The majority of CDK inhibitors bind to the ATP pocket as ATP-competitive inhibitors with essential structural hydrogen-bonding motifs^28^. Cyclin-dependent kinase 2 (CDK2) belongs to the serine/threonine protein kinase family, and the CDK2 activity is found to be typically high in different types of human cancers. Studies have reported that CDK2 overexpression indicates poor prognosis in patients with HCC, and inhibition of CDK2 activity could reverse the malignant phenotype of cancer cells. Many studies revealed a key role of CDK2 in EGF-induced cell transformation and the associated signal transduction pathways^29^. The literature survey revealed that EGFR and CDK2 were the key targets for many antitumor agents e.g. cinobufagin^30^. In addition, biological and computational evidence supported that anticancer agents such as benzamide-substituted chalcones exerted their anti-proliferative effects via dual EGFR/CDK2 inhibitory activities^31^.

It is worth mentioning that, dual-specificity tyrosine phosphorylation-regulated kinase 3 (DYRK3) is a regulator of phase transition during mitosis^32^. Furthermore, it is able to promote mTORC1 activity, which is associated with resistance to EGFR-mediated endocrine therapy, and other forms of targeted therapy, also it regulates fundamental cellular functions including transcription, translation, proliferation, growth and survival^33^. Moreover, Glycogen synthase kinase 3 (GSK3) is involved in modulating numerous signalling pathways affecting metabolism, tumorigenesis, proliferation, apoptosis, autophagy, development, and differentiation involved in metabolic regulation^34^. GSK3 inhibitors were reported to suppress breast tumour growth in pre-clinical models^35^^,^^36^.

**Figure 1.**
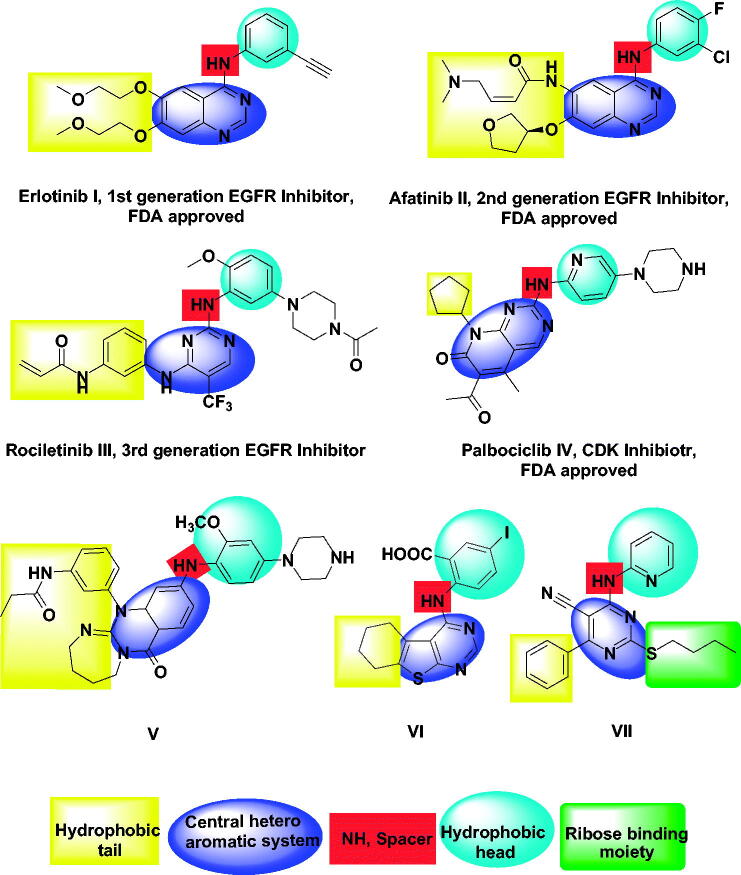
FDA approved and reported kinases inhibitors with their essential pharmacophoric features.

Until now, there are many FDA-approved drugs for the treatment of cancer targeting EGFR as erlotinib **I** and afatinib **II**. The first generation of these inhibitors (as erlotinib **I**) gave good benefits in the treatment of non-small-cell lung cancer^37^. The second generation of EGFR inhibitors (as afatinib **II**) was approved to overcome EGFR mutation-related resistance^38^. The third generation of EGFR inhibitors (as rociletinib **III**) had overcome induced toxicity caused by 2^nd^ generation^39–41^. Palbociclib **IV** is the first FDA-approved cyclin-dependent kinase inhibitor that acts by binding to the ATP pocket with an IC_50_ in the range of 9–15 nM^27^ (Figure 1).

**Figure 2.**
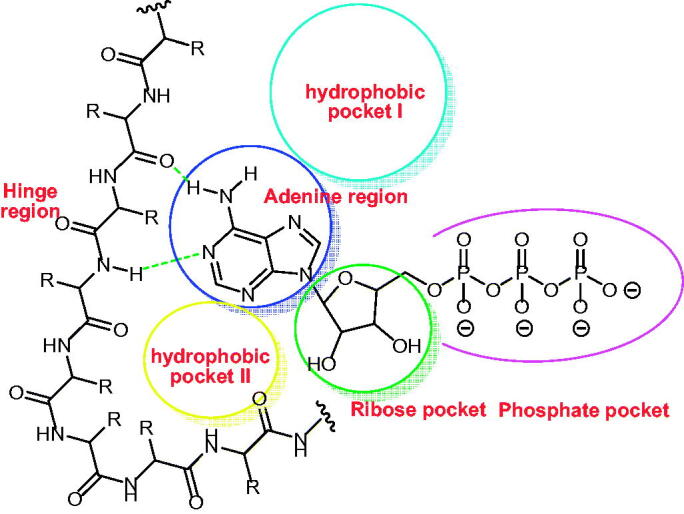
ATP binding site of EGFR cavity composed of five main parts^47–49^.

EGFR inhibitors have some pharmacophoric features which are essential for maximal affinity against the ATP binding site of EGFR. These features include i) a flat hetero aromatic ring system that occupies the adenine binding pocket^42^, ii) terminal hydrophobic head which can occupy the hydrophobic region I, iii) imino group (NH, spacer) which can occupy the space between the adenine binding region and the hydrophobic region I^43^, iv) hydrophobic tail which occupies the hydrophobic region II^44^^,^^45^, ribose binding moiety which can occupy the ribose binding pocket. Till now, there are limitations in research that target the ribose binding pocket^46^ (Figure 2).

Privileged structures of diazepine have been widely used as effective templates for anticancer agents especially targeting EGFR enzyme^50^. Xu et al., described in 2013 a compound with a 1,3‐diazepine moiety (compound **V**) presenting a potent inhibition activity against the EGFR790M mutant (IC_50_ = 3.36 nM) and the double EGFRL858R/T790M mutant (IC_50_ = 1.69 nM)^51^. Also, in 2019, a series of thieno[2,3-*d*]pyrimidine derivatives were designed and synthesised as EGFR and HER2 tyrosine kinase inhibitors. Compound **VI** exhibited promising cytotoxic activity^47^. In 2020, a new series of pyrimidine-5-carbonitrile derivatives were designed and synthesised as EGFR inhibitors. Compound **VII** showed high inhibitory activities against EGFR^WT^ and EGFR^T790M^^48^ (Figure 1).

**Figure 3.**
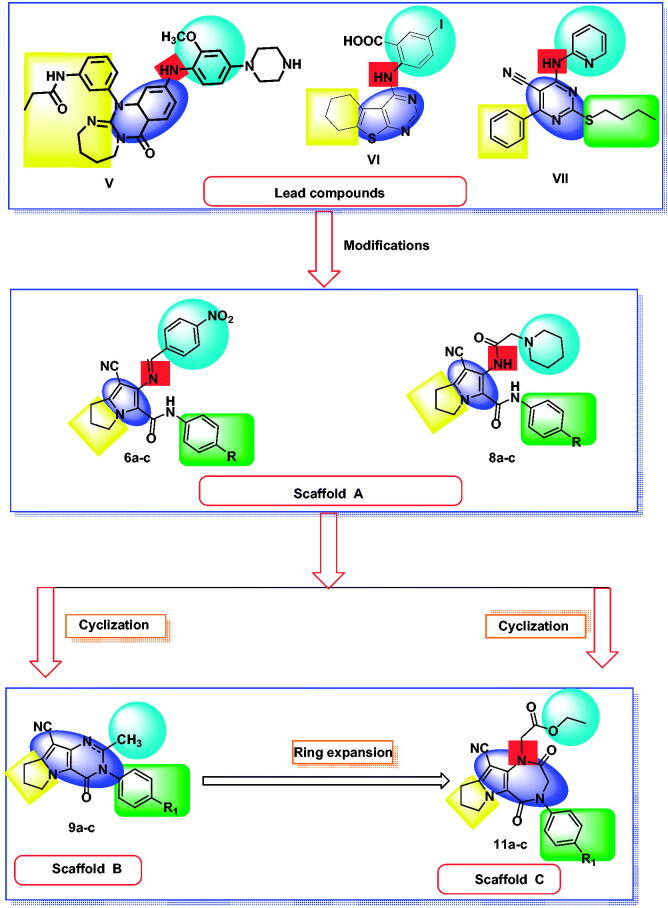
Design strategies of Scaffolds **A**, **B** and **C**.

Based on the mentioned facts and as an extension of the previous efforts in the design and synthesis of new anticancer agents^48^^,^^52–59^ especially to target EGFR^47^^,^^48^^,^^60^ we used the 1*H*-pyrrole, pyrrolo[3,2-*d*]pyrimidine, and pyrrolo[3,2-*e*][1, 4]diazepine moieties as building blocks for the design and synthesis of new EGFR and CDK inhibitors (Figure 3).

### 1.1. Rationale of molecular design

In the present work, the previously active compounds (**V, VI** and **VII)** were used as lead compounds to design new EGFR inhibitors. Dramatic modifications were achieved to reach more promising active candidates against EGFR and CDK. The modifications were performed on four features. The flat hetero aromatic system was modified to be 1*H*-pyrrole (compounds **6a–c** and **8a–c**) (Scaffold **A)**, pyrrolo[3,2-*d*]pyrimidine (**9a–c**) (Scaffold **B)**, and pyrrolo[3,2-*e*][1, 4]diazepine (compounds **11a–c**) (Scaffold **C)**. ii) For the terminal hydrophobic head, we used different hydrophobic moieties as substituted phenyl groups (compounds **6a–c**) or aliphatic moieties (compounds **8a–c, 9a–c,** and **11a–c**). Regarding the hydrophobic tail, we used the pyrrolidine moiety in all the designed compounds. The NH spacer was kept as it is in compounds **8a–c**, whereas it was changed into -N = moiety in compounds **6a–c**, and deleted in compounds **9a–c** relaying on the two nitrogen atoms of pyrimidine moiety to act as hydrogen bond centres. Moreover, in compounds **11a–c**, it was modified to be inside the ring structure as a heteroatom. To occupy ribose binding moieties, we used different aromatic structures. Different structure modifications were then achieved to obtain a SAR study as a second aim in our work.

To confirm our rationale, the synthesised compounds were investigated for their antiproliferative activities against a panel of human cancer cell lines (Hep3B, HCT116, and MCF-7). In addition, the most active anti-proliferative agent was further subjected to a Kinase profiling test to assess its activity against EGFR, CDK, and other kinases. Furthermore, to reach a good insight into the activity of the most active candidate at the molecular level, cell cycle analysis was carried out. Finally, *in silico*, docking, ADMET, and toxicity studies were performed to predict the possible binding interaction of the synthesised compounds against the prospective targets (EGFR and CDK) as well as to calculate the proposed kinetic and toxicity profile.

## 2. Results and discussion

### 2.1. Chemistry

**Scheme 1.**
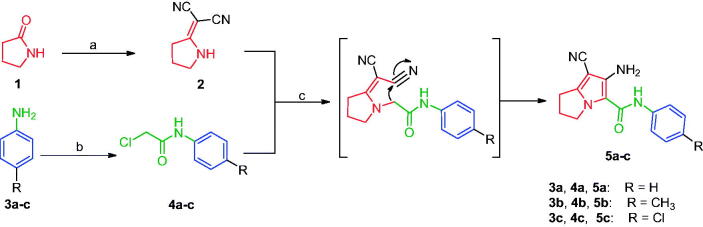
Construction of compounds **5a–c**. Reagents and conditions: (a) (CH_3_)_2_SO_4_, benzene, CH_2_(CN)_2_, reflux, 6 h; (b) ClCH_2_COCl, glacial acetic acid, CH_2_COONa, 30–40 °C, 2 h; (c) acetone, K_2_CO_3_, reflux, 24 h.

**Scheme 2.**
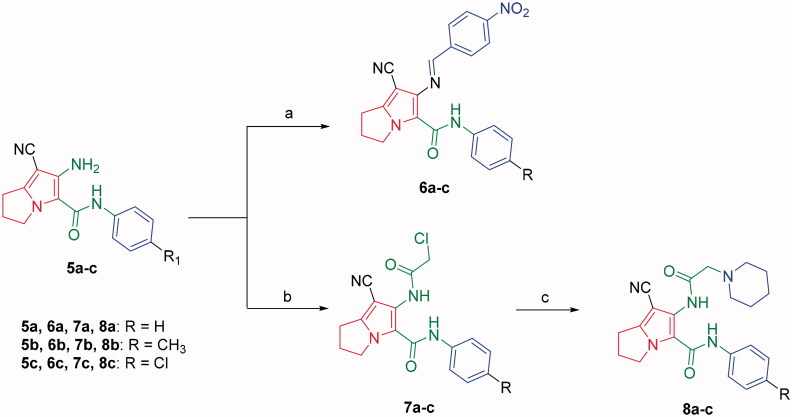
Synthesis of compounds **6a–c**, **7a–c** and **8a–c**. Reagents and conditions: (a) 4-Nitrobenzaldehyde, absolute ethanol, glacial acetic acid; (b) ClCH_2_COCl, benzene; r.t.; 48 h; (c) piperidine, NaHCO_3_, absolute ethanol, reflux, 8 h.

Compounds **8**^61^ and **10a–c**^62^ were synthesised according to reported methods, which were then used to afford the intermediates **11a–c** according to the reported procedure^63^. Condensation of compounds **5a–c** (Scheme 1) with 4-nitrobenzaldehyde in absolute ethanol afforded three Schiff base derivatives (**6a–c**, Scheme 2). The IR spectra of compounds **6a–c** showed sharp absorption bands at 2210–2214 cm^−1^_,_ indicating the cyano group. Furthermore, the ^1^H NMR spectra of compounds **6a–c** showed two doublet signals at a range of 8.10–8.42 ppm due to the aromatic protons of the 4-nitrobenzylidene moiety. Moreover, a singlet signal appeared at a range of 9.28–9.31 corresponding to the N = CH proton. While their ^13 ^C NMR spectra revealed two signals at a range of 156.3–158 ppm indicating the N=CH and the carbonyl carbon in each of the three derivatives. The free amino group of compounds **5a–c** was further acylated with 2-chloroacetyl chloride in benzene to give compounds **7a–c**. Compounds **8a–c** were prepared by *N*-alkylation of piperidine with the terminal chlorinated side chain of **7a–c** in absolute ethanol, using sodium bicarbonate to neutralise the hydrogen chloride liberated from this reaction (Scheme 2). The ^1^H NMR spectra of compounds **8a–c** confirmed the aliphatic protons of the piperidin-1-yl ring; multiplet and triplet signals at a range of 1.50–2.74 ppm. Their ^13 ^C NMR spectra further revealed three additional signals in the range of 23.4–55.0 ppm due to the aliphatic carbons of the piperidin-1-yl ring.

**Scheme 3.**
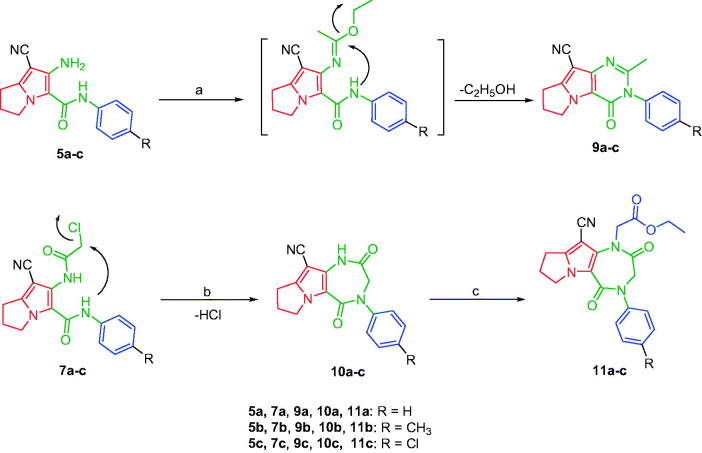
Synthesis of compounds **9a–c**, **10a–c** and **11a–c**. Reagents and conditions: (a) Excess CH_3_C(OC_2_H_5_)_3_, reflux, 12 h; (b) K_2_CO_3_, DMF, r.t., 48 h; (c) ClCH_2_COOC_2_H_5_, K_2_CO_3_, acetone, reflux, 6 h.

Moreover, several studies reported the use of triethyl orthoformate to produce cyclized derivatives^64^. The tricyclic pyrrolo[3,2-*d*]pyrimidine derivatives **9a–c** were achieved by refluxing compounds **5a–c** with excess triethyl orthoformate (Scheme 3). The NHs stretching bands disappeared in the IR spectra of compounds **9a–c**. Further, the ^1^H NMR spectra of compounds **9a–c** confirmed disappearance of the exchangeable protons’ signals, corresponding to compounds **5a–c** NHs protons. Additionally, they showed singlet signals at a range of 2.25–2.27 ppm corresponding to the aliphatic methyl protons. Furthermore, the ^13 ^C NMR spectra revealed the presence of two additional signals; the first one at the range of 24.3–24.4 ppm due to the aliphatic methyl group at C-2 and a second signal at the range of 153.7–154.1 ppm assigned for the C-2. Whereas, the pyrrolo[3,2-*e*][1, 4]diazepine derivatives **10a–c** were obtained by intramolecular cyclisation of compounds **7a–c** using potassium carbonate in dimethylformamide (Scheme 3). Finally, *N*-alkylation of compounds **10a–c** with ethyl chloroacetate afforded the ethyl ester derivatives **11a–c** using potassium carbonate in acetone (Scheme 3). The disappearance of the amidic protons (CONH) of the starting materials **10a–c** was observed in the ^1^H NMR spectra of compounds **11a–c**, in addition to the presence of triplet signals at 1.28–1.29 ppm and quartette signals around 4.24 ppm due to the ethyl group. The ^13 ^C NMR spectra of compounds **11a–c** revealed three signals in the range of 159.6–167.6 ppm that were assigned for the carbon atoms of the three carbonyl groups.

### 2.2. Biological evaluation

#### 2.2.1. Anticancer activity

Evaluation of the anticancer activity was performed against liver (Hep3B), colon (HCT116), and breast (MCF-7) cancer cell lines in the Centre of Genetic Engineering, Al-Azhar University, Cairo, Egypt, using Sulforhodamine-B (SRB) assay^65^. Doxorubicin (DOX) was used as a reference drug. The survival curve was obtained by plotting concentrations of the compound under investigation against the survival fraction of the tumour cells. Then, results were expressed in half maximal inhibitory concentration (IC_50_).

All the tested derivatives showed potent antiproliferative activities (IC_50_ = 0.009 − 2.195 µM) against the three cancer cell lines (Table 1). A closer look at the results revealed that compound **8b** exhibited the highest cytotoxic activity against Hep3B and MCF-7 cell lines with IC_50_ values of 0.049 and 0.043 µM, respectively. On the other hand, compound **8b** displayed the highest cytotoxic activity against the HCT116 cell line with IC_50_ values of 0.011 µM.

**Table 1.** IC_50_ values of the new compounds (**6a–c, 8a–c, 9a–c,** and **11a–c**) against Hep3B, HCT116 and MCF-7 cell lines.

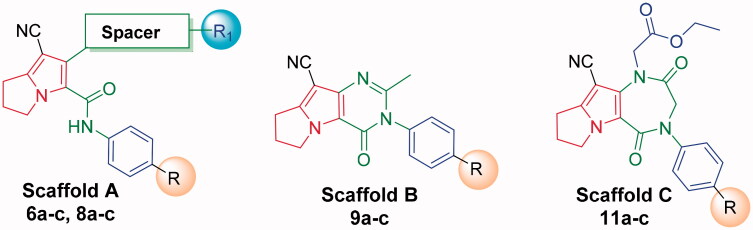							
Comp.	Scaffold	R	Spacer	R_1_	IC_50_ (µM)		
					Hep3B	HCT116	MCF-7
**6a**	A	H	N = CH-	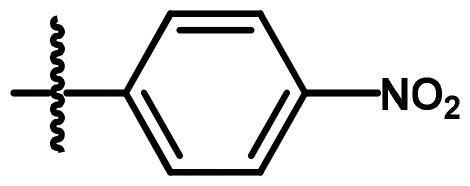	0.219	0.213	0.422
**6b**	A	CH_3_	N = CH-	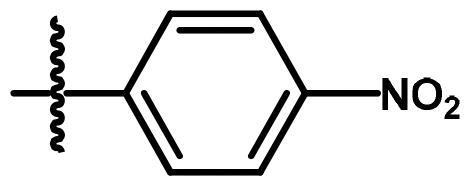	1.956	2.069	0.336
**6c**	A	Cl	N = CH-	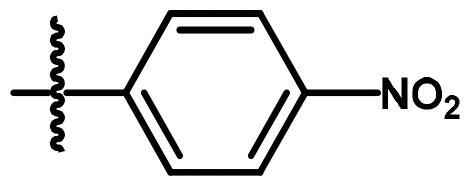	1.470	0.955	2.195
**8a**	A	H	-NHCOCH_2_-	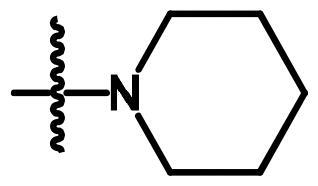	0.313	0.408	0.315
**8b**	A	CH_3_	-NHCOCH_2_-	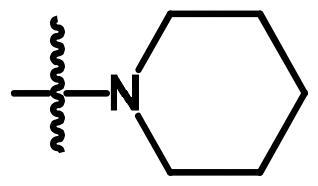	0.049	0.031	0.043
**8c**	A	Cl	-NHCOCH_2_-	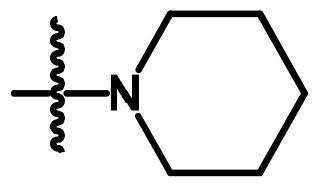	0.144	0.180	0.294
**9a**	B	H	–	–	0.487	0.011	0.137
**9b**	B	CH_3_	–	–	0.265	0.820	0.644
**9c**	B	Cl	–	–	0.072	0.009	1.479
**11a**	C	H	–	–	0.218	0.840	0.154
**11b**	C	CH_3_	–	–	0.271	0.303	0.354
**11c**	C	Cl	–	–	0.179	0.286	0.364
**DOX**	**-**	**-**	**-**	–	0.005	0.008	0.008

Hep3B, Liver cancer cell line; HCT116, colon cancer cell line; MCF-7, breast cancer cell line; DOX, doxorubicin.

##### 2.2.2.1. Structure-Activity Relationship (SAR)

Inspecting the results of the anti-proliferative activity of the designed candidates, we concluded a valuable SAR. With reference to their cytotoxic activity, it was noticed that counterparts incorporating 2,3-dihydro-1*H*-pyrrolizine moiety (scaffold A) were slightly more advantageous than the 3*H*-pyrimido[4,5-b]pyrrolizine derivatives (scaffold B). However, hexahydro-[1, 4]diazepino[5,6-b]pyrrolizin containing derivatives (scaffold C) displayed less potent inhibitory activity against the tested cell lines.

Concerning the di-aryl compounds **6a–c**, compound **6a **(unsubstituted-*N*-phenylcarbamoyl derivatives) displayed higher activity than **6b** and **6c** (4-methyl and 4-chloro-*N*-phenylcarbamoyl derivatives, respectively) against Hep3B (IC_50_ = 0.219 µM) and HCT116 (IC_50_ = 0.213 µM) cancer cell lines. While the methyl analogue **6b** was the most active member against MCF-7 cell line with IC_50_ value of 0.336 µM. Replacement of the aromatic 6–4-nitrobenzylidene)amino side chain in compounds **6a–c** by the 2-(piperidin-1-yl)acetamido moiety (compounds **8a–c**) resulted in a slightly enhanced cytotoxicity; compounds **8a–c** showed IC_50_ values in the range of 0.031–0.408 µM against the three cell lines. Among the three derivatives **8a–c**, the methyl derivative **8b** displayed the highest anticancer activity against the three cell lines.

Evaluation of the anticancer activity of pyrrolo[3,2-*d*]pyrimidine **9a–c**, revealed that the 4-chloro congener derivative **9c** was the most active against HCT116 cell line among all the tested compounds (IC_50_ = 0.009 µM). While, the pyrrolo[3,2-*e*][1, 4]diazepine derivatives (compounds **11a–c**) showed comparable activity to their fused pyrrolo[3,2-d]pyrimidine analogues (**9a–c**) with enhancement in activity of the 4-chloro **11c** over **9c** against MCF-7 cell line (IC_50_ = 0.364 versus 1.479 µM). Thus, the SAR study pointed out the significance of a single methyl group as it presents in the most active compounds **8b** (*N*-*p*-tolylcarbamoyl derivative) and **9a,c**; 2-methyl group on the fused pyrrolo[3,2-*d*]pyrimidine scaffold. However, the comparable activity among the three series (**A–C** scaffolds) is raising a query about the significance of the molecular modifications on the physicochemical properties, which could affect the cellular barriers positively.

#### 2.2.2. EGFR and CDK-2 inhibitory activity

According to the anti-proliferative activity of the tested compounds, **8b** showed a substantial broad-spectrum activity against Hep3B, HCT116 and MCF-7 cell lines (IC_50_ = 0.049, 0.031 and 0.043 µM, respectively). Thus, compound **8b** was tested for its inhibitory activity against both EGFR and CDK-2.

The results revealed that compound **8b** showed excellent activity against CDK2 with an inhibition value of 15% compared to the reference molecule, imatinib (2%). On the other hand, it showed less activity against EGFR (% Inhibition = −70%) compared to imatinib (% Inhibition = −13%) (Table 2).

**Table 2.** Inhibitory activity of compound **8b** and imatinib against 20 kinases at 10 µM^66^.

Kinase	% Inhibition		Kinase	% Inhibition	
	Imatinib*^a^*	8b*^b^*		Imatinib*^a^*	8b*^b^*
AMPK (A1/B1/G1)	−14%	−18%	EPHA1	−22%	−46%
ALK1	−18%	−21%	FLT1	10%	−8%
ASK1	−5%	−23%	GRK1	−2%	−1%
Aurora A	−38%	−6%	GSK3 alpha	1%	10%
BLK	−3%	−36%	MSK1	−49%	−48%
BRAF	−13%	−77%	NEK1	2%	−8%
CDK2/Cyclin A1	2%	15%	p38 Alpha	−17%	−23%
CK1 Alpha 1	0%	−10%	PDK1	−34%	−28%
DYRK3	10%	23%	PRKG1	−4%	−4%
EGFR	−13%	−70%	SGK1	−3%	−10%

The + ve values indicate the inhibition % and − ve values indicate increase in enzyme activity.

#### 2.2.3. Kinase profiling test

The previous results encouraged us to test compound **8b** against other different kinases (18 kinases) to reach a good insight into its kinases inhibitory profile. The result showed that compound **8b** has good inhibitory activity against DYRK3 and GSK3 alpha kinases. For its activity against DYRK3, it showed an inhibition value of 23% compared to imatinib (% Inhibition = 10%). Regarding its activity against GSK3 alpha, it showed an inhibition value of 10% compared to imatinib (% Inhibition = 1%).

Additionally, compound **8b** showed imatinib comparable activity against ALK1, AMPK (A1/B1/G1), GRK1, MSK1, p38 Alpha, PDK1, and PRKG1. On the other hand, it showed moderate to weak activity against CK1 Alpha 1, Aurora A, BLK, BRAF, ASK1, EPHA1, FLT1, NEK1, and SGK1 (Table 2).

#### 2.2.4. Determination of cell cycle perturbations

**Figure 4.**
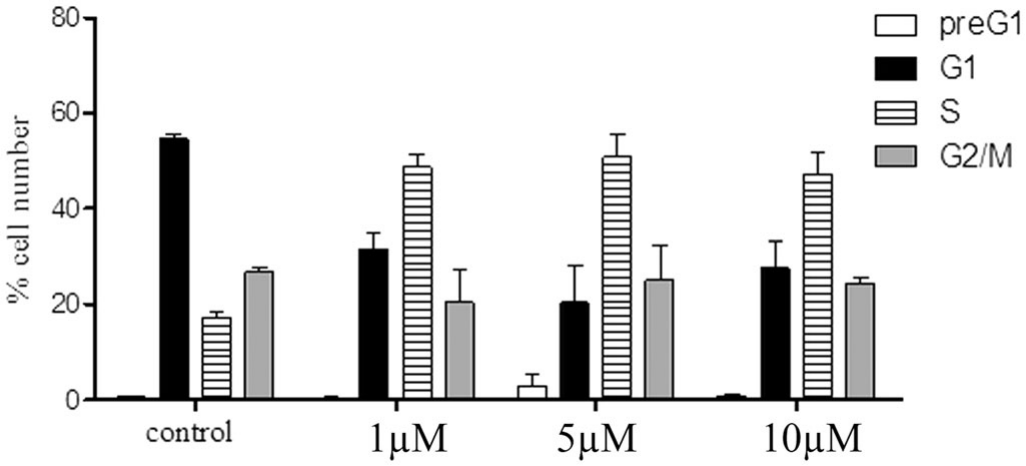
Cell cycle distribution of MCF-7 treated with compound **6a** (µM, 72 h: x axis); % cell (y axis).

**Figure 5.**
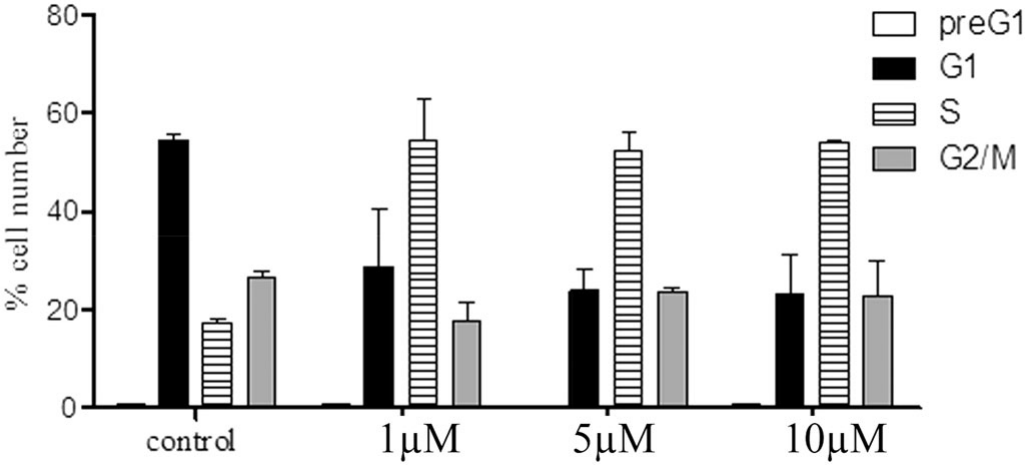
Cell cycle distribution of MCF-7 treated with compound **8b** (µM, 72 h: x axis); % cell (y axis).

To study the mechanistic actions regarding compounds **6a** and **8b**, cell cycle analyses using flow cytometry (BC, FC500) were performed. The results of the cell cycle perturbation of the MCF-7 cell line treated with compounds **6a** and **8b** (72 h, separately) were presented in Figures 4 and 5, respectively. Each of the two compounds caused a three-fold increase of cells in the S phase at 1 µM compared to control. This increase in the S-phase cell population was accompanied by a concomitant decrease in the G_1_ cell population. However, there was no considerable change in the S phase cellular population at 5 and 10 µM, which means that the effect of compounds **6a** and **8b** are not dose-dependent.

### 2.3. *In silico* studies

#### 2.3.1. Docking studies

A docking study was conducted in the hopes of learning more about how the synthesised compounds interact with their targets^67–69^. EGFR (PDB: 4HJO), CDK-2 (PDB: 6GUH), DYRK3 (PDB: 5Y86), and GSK3 (PDB: 5HLP) were employed as biological targets in docking investigations utilising MOE 14.0 software. The co-crystallised ligands (erlotinib and AZD5438) were utilised as anti-EGFR and anti-CDK-2 reference compounds respectively. When compared to the reference molecules, the docking results demonstrated. that the synthesised compounds have a high affinity for the two examined targets (Table 3).

**Table 3.** The binding free energies of the synthesised compounds against EGFR and CDK-2.

Comp.	Binding free energy (kcal/mol)	
	EGFR	CDK-2
**6a**	−18.33	−23.67
**6b**	−18.97	−23.84
**6c**	−19.14	−23.60
**8a**	−20.67	−24.89
**8b**	−22.46	−24.52
**8c**	−20.08	−24.20
**9a**	−17.69	−17.77
**9b**	−18.90	−17.74
**9c**	−17.72	−16.71
**11a**	−21.54	−24.08
**11b**	−23.30	−24.18
**11c**	−18.93	−23.98
**Erlotinib**	−23.49	–
**AZD5438**	–	−21.66

**Figure 6.**
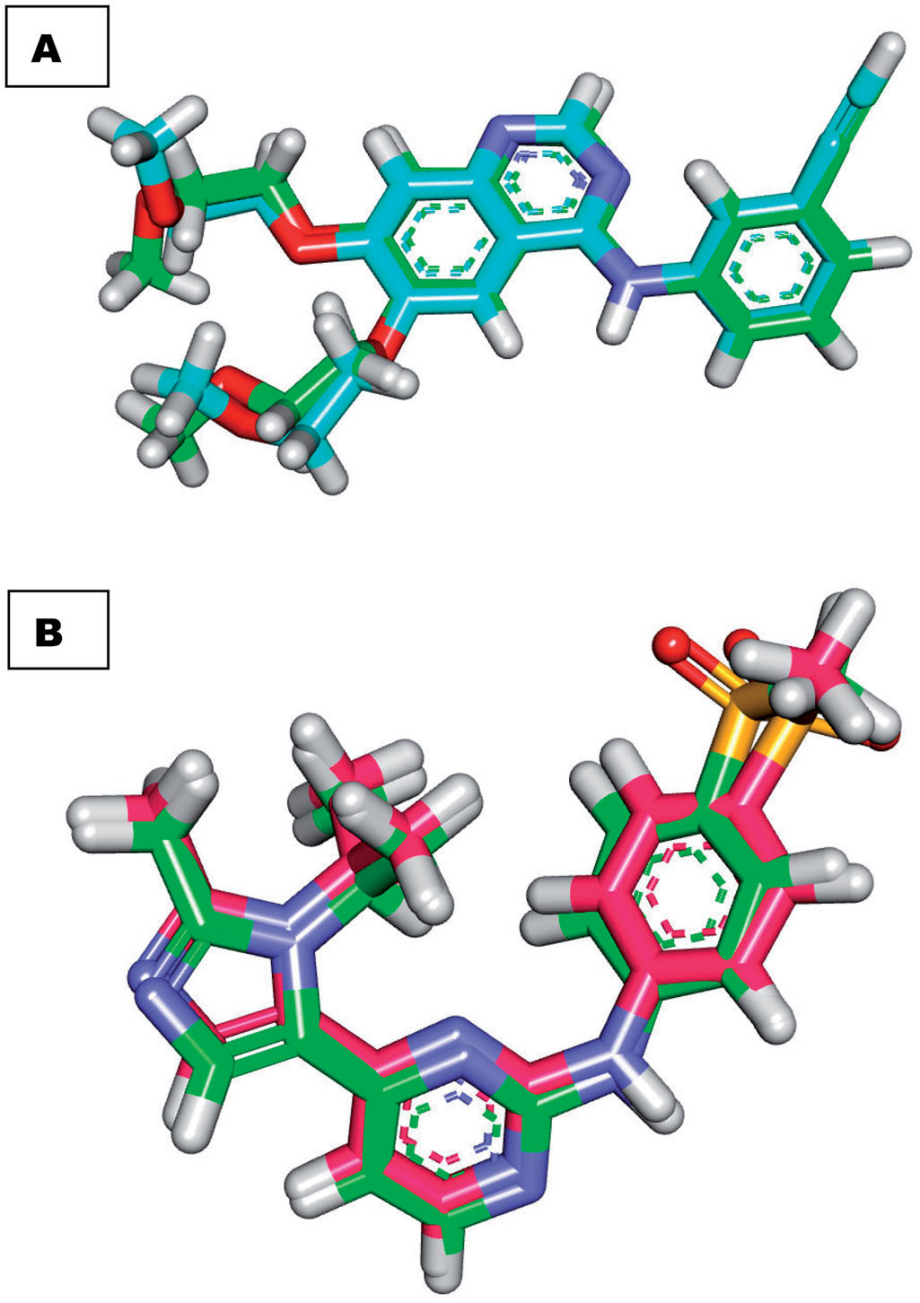
(A) superimposition of the docked ligand of erlotinib (turquoise) and the original ligand (green) with an RMSD value of 0.88 Å. (B) superimposition of the docked ligand of AZD5438 (pink) and the original ligand (green) with RMSD value of 0.54 Å.

Redocking of co-crystallised ligands (Erlotinib and AZD5438) against EGFR and CDK-2, respectively, was used to validate docking experiments. Erlotinib and AZD5438 had RMSDs of 0.88 and 0.54 Å for docked and original ligands, respectively. The validity of the docking operation is shown by these values (Figure 6).

**Figure 7.**
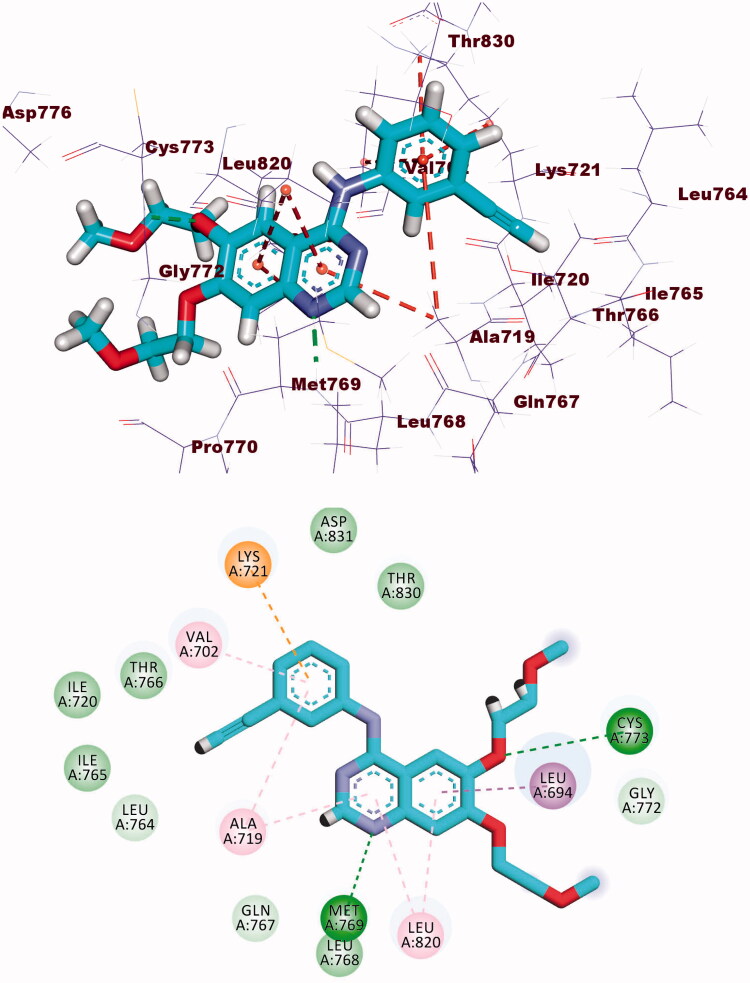
Erlotinib docked into the active site of EGFR.

The binding energy of erlotinib was found to be −23.49 kcal/mol. The quinazoline molecule was buried in the adenine pocket, creating a hydrogen connection with Met769. Furthermore, Lue694, Ala719, and Leu820 established four hydrophobic contacts with quinazoline. The ethynylphenyl moiety was positioned in the hydrophobic pocket I, resulting in three hydrophobic interactions with Ala719, Val702, and Lys721. The 2-methoxyethoxy groups formed a hydrogen bond with Cys773 in the hydrophobic region II (Figure 7).

**Figure 8.**
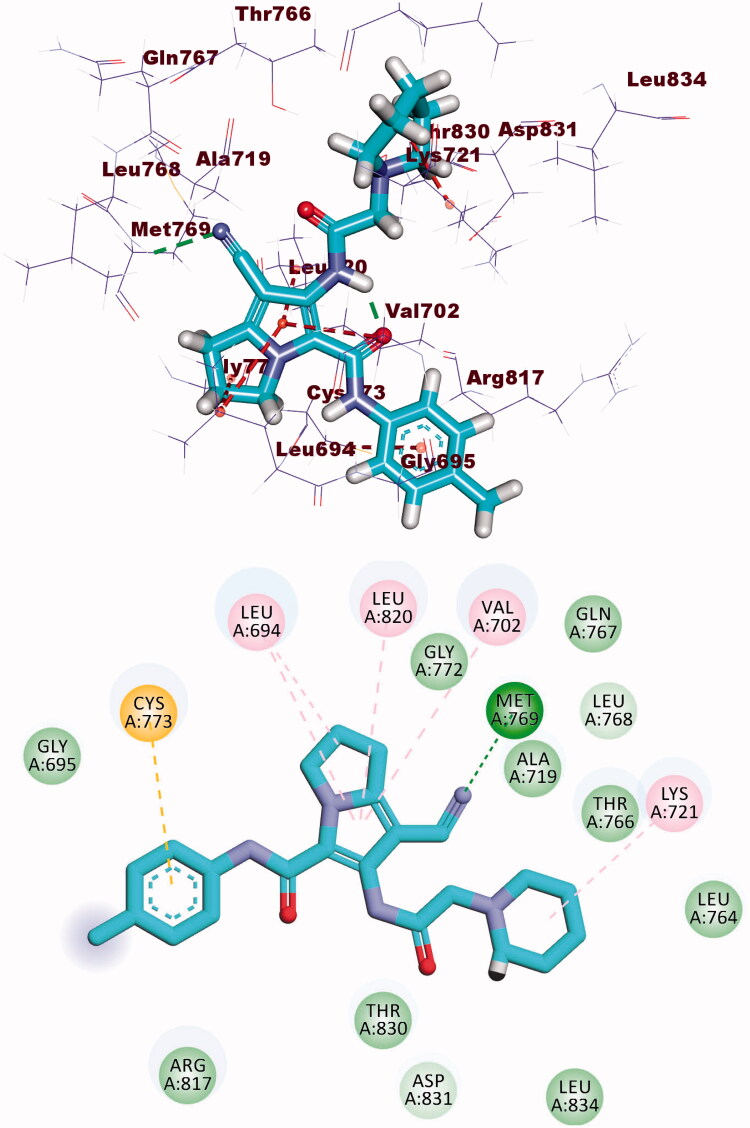
Compound **8b** docked into the active site of EGFR.

Compound **8b** showed a binding mode like that of erlotinib with a binding energy of −22.46 kcal/mol. The 1*H*-pyrrole-3-carbonitrile moiety occupied the adenine pocket of the EGFR forming three hydrophobic interactions with Leu820, Leu694, and Val702. Piperidine moiety was oriented into the hydrophobic pocket I to form one hydrophobic interaction with Leu694. The pyrrolidine moiety occupied the hydrophobic pocket II forming one hydrophobic interaction with Lys721 in close contact with Thr766, Leu764, and Leu834. Moreover, *p*-tolyl moiety occupied the ribose binding pocket forming one electrostatic attraction with Cys773 (Figure 8).

**Figure 9.**
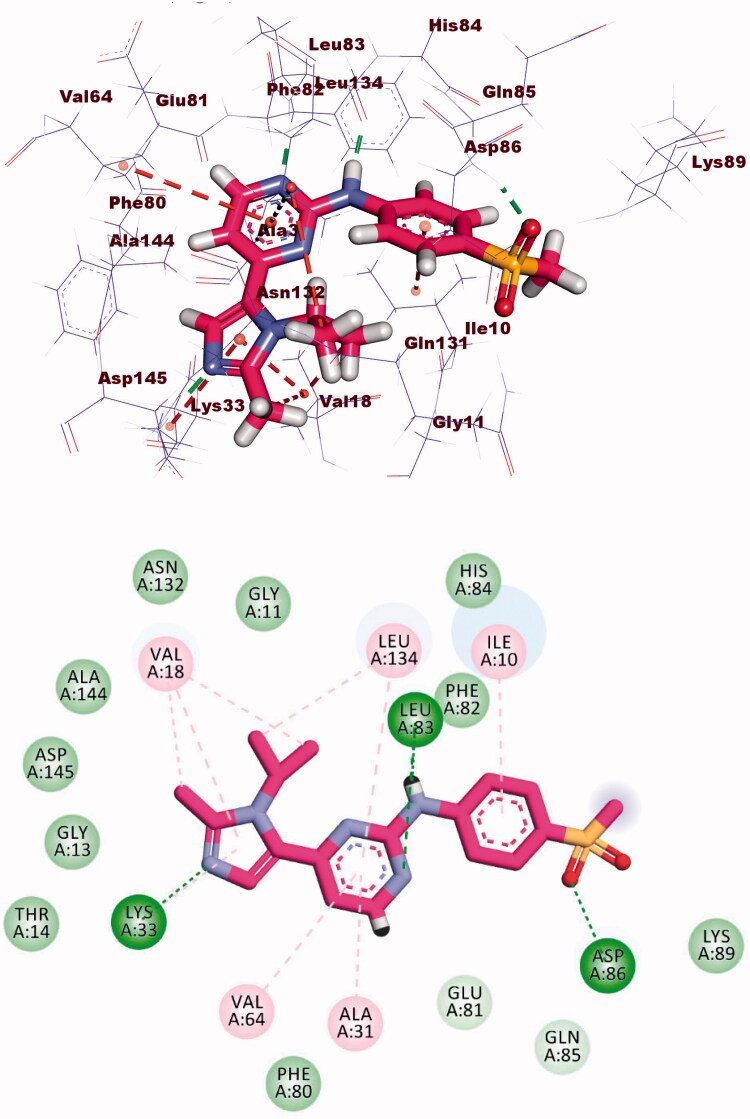
Co-crystallised ligand (AZD5438) docked into the active site of CDK-2.

The co-crystallised ligand (AZD5438) showed binding energy of −21.66 kcal/mol against CDK-2. The pyrimidine moiety occupied the adenine pocket forming three hydrophobic bonds with Leu134, and Ala31, Val64. The NH-linker formed one hydrogen bond with Leu83. The terminal methylsulfonyl benzene moiety occupied the hydrophobic pocket forming a hydrogen bond with Asp86 and one hydrophobic interaction with Ile10. Additionally, the 1-isopropyl-2-methyl-1*H*-imidazole moiety occupied the other hydrophobic region forming a hydrogen bond with Lys33. Also, it formed four hydrophobic interactions with Leu134 and Val18 (Figure 9).

**Figure 10.**
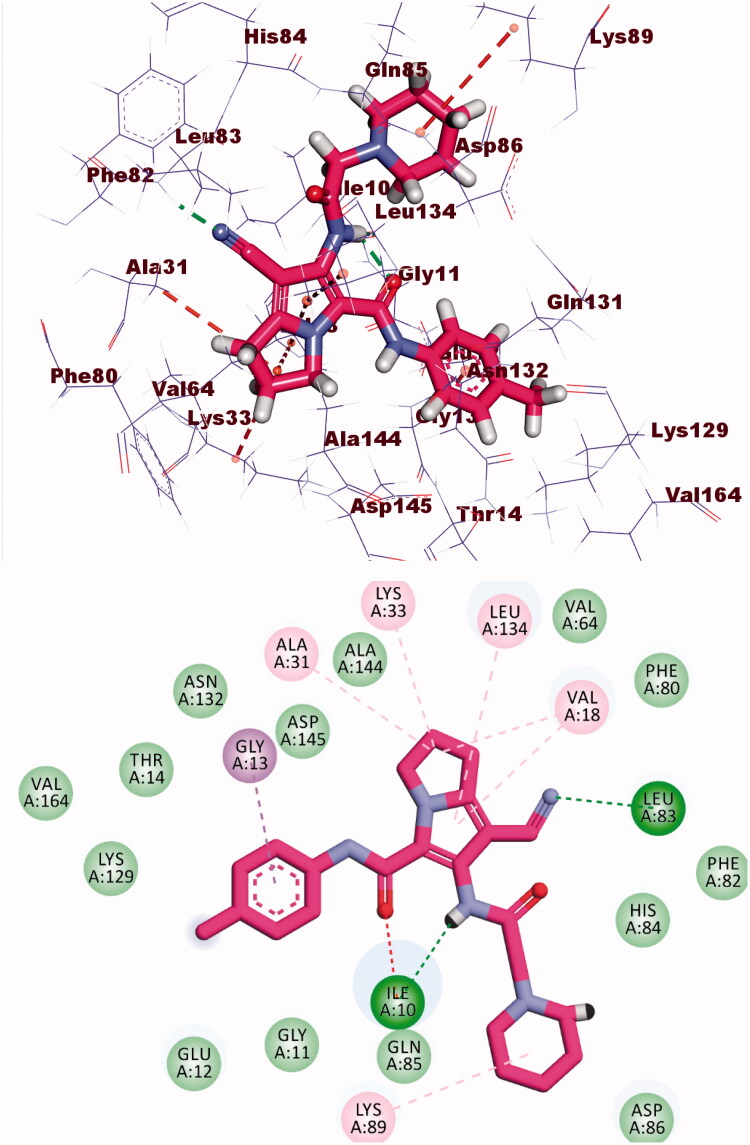
(A) Binding of compound **8b** with CDK-2.

Compound **8b** showed a binding mode like that of AZD543 with a binding energy of −24.52 kcal/mol. The 1*H*-pyrrole-3-carbonitrile moiety occupied the adenine pocket forming two hydrophobic interactions with Leu134 and Val18. The pyrrolidine moiety was oriented into the hydrophobic pocket forming three hydrophobic interactions with Val18, Lys33, and Ala31. The piperidine moiety occupied another hydrophobic pocket forming one hydrophobic interaction with Lys89 in close contact with Asp86 and Gln85. The two amide linkers formed one hydrogen bond and one electrostatic interaction with Ile10. Moreover, *p*-tolyl moiety occupied the ribose binding pocket forming one hydrophobic interaction with Gly13 (Figure 10).

**Figure 11.**
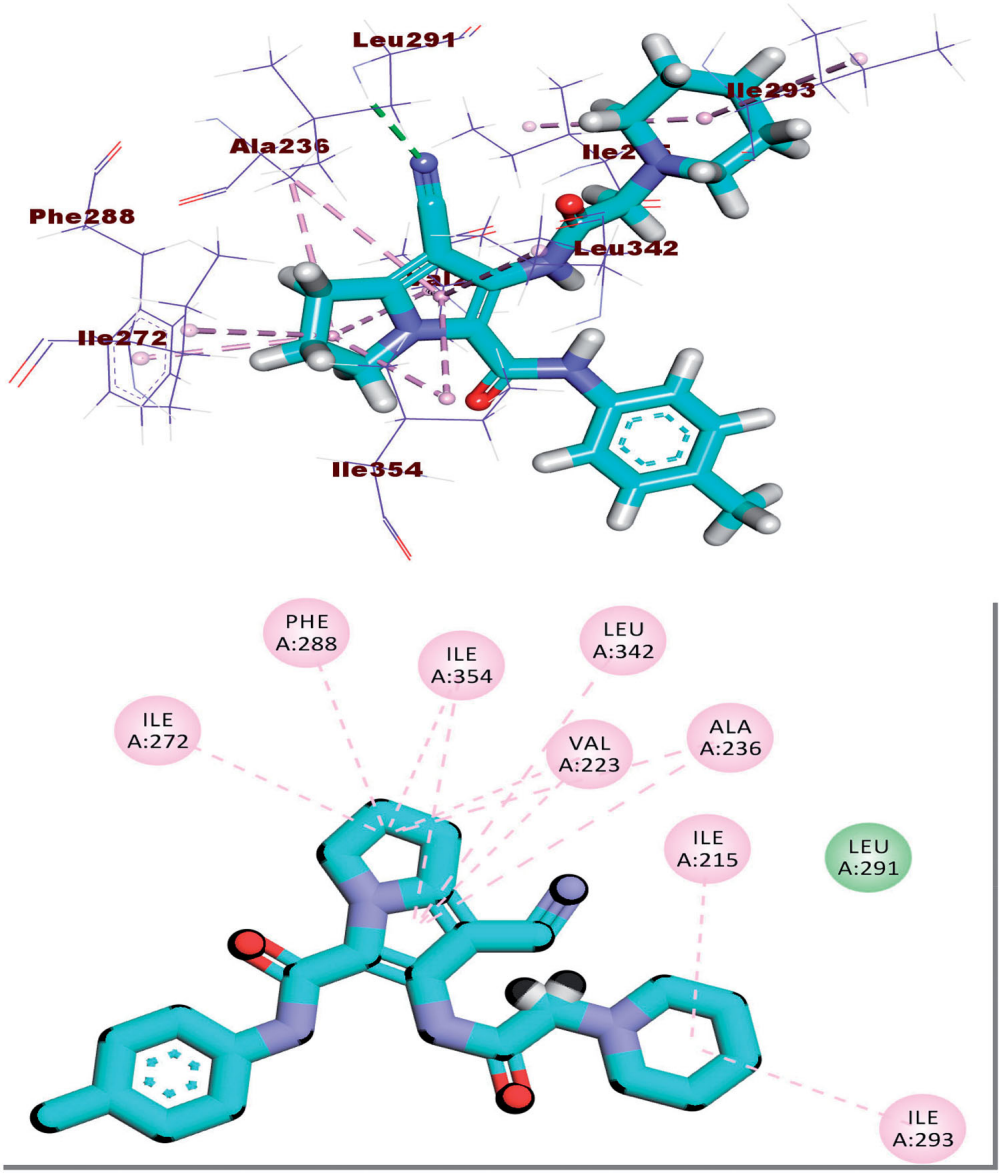
(A) Binding of compound **8b** with DYRK3.

**Figure 12.**
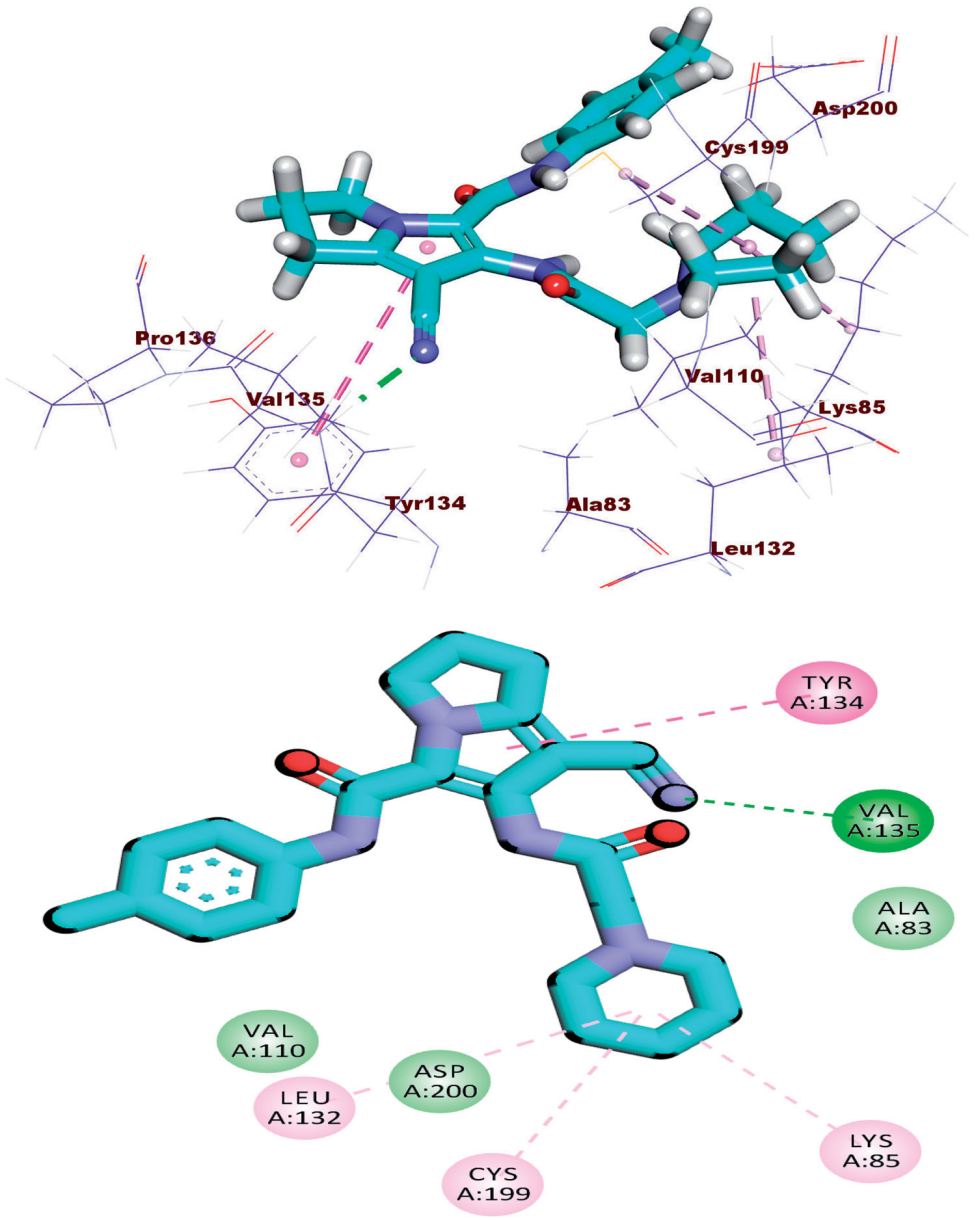
(A) Binding of compound **8b** with GSK3 alpha.

Docking calculations between compound **8b** and DYRK3 and GSK3 revealed that it could occupy the active sites and interact with the key amino acids of each enzyme (Figure 11 and Figure 12) with the binding energy of −24.54 and −16.85 kcal/mol, respectively. The binding free energy of the co-crystallised ligands of DYRK3 (HRM) and GSK3 (65 A) was calculated to be −20.80 and −16.41 kcal/mol, respectively and the binding modes were presented in Supplementary data.

#### 2.3.2. *In silico* toxicity prediction

In this work, eight toxicity parameters were estimated computationally depending on the constructed toxicity models in Discovery studio software^70–72^. The results revealed that the calculated toxicity potential of the synthesised compound was low. Except for compound **6a**, all compounds were non-carcinogenic for mouse females based on the FDA rodent carcinogenicity model. In addition, compounds **6a, 8a, 8b, 9a,** and **11a** showed carcinogenic potency TD_50_ values ranging from 11.774 to 71.541 mg/kg body weight/day, which were higher than that of erlotinib (8.057 mg/kg body weight/day). All compounds had rat maximum tolerated dose values less than that of erlotinib (0.083 g/kg body weight) except compound **8c** (0.107 g/kg body weight). For the rat oral LD_50_ model, except compounds **9a** and **9c,** all compounds showed oral LD_50_ values higher than that of erlotinib (0.662 mg/kg body weight/day). In addition, all compounds exhibited rat chronic LOAEL values higher than that of erlotinib **(**0.036 mg/kg body weight/day) except compounds **8c** and **11c**. Moreover, all the tested compounds were predicted to be mild irritants in the ocular irritancy model, and non-irritants in the skin irritancy model (Table 4).

**Table 4.** Toxicity properties of the synthesised compounds.

Comp.	FDA rodent carcinogenicity (mouse-female)	Carcinogenic potency TD_50_ (Rat)^a^	Rat maximum tolerated dose (feed)^b^	Rat oral LD_50_^b^	Rat chronic LOAEL^b^	Ocular irritancy	Skin irritancy
**6a**	Single-Carcinogen	18.143	0.060	0.800	0.157	Irritant	Non-Irritant
**6b**	Non-Carcinogen	2.356	0.049	2.050	0.096	Irritant	Non-Irritant
**6c**	Non-Carcinogen	1.839	0.073	0.838	0.063	Irritant	Non-Irritant
**8a**	Non-Carcinogen	71.541	0.087	1.162	0.071	Irritant	Non-Irritant
**8b**	Non-Carcinogen	11.813	0.072	3.451	0.054	Irritant	Non-Irritant
**8c**	Non-Carcinogen	7.264	0.107	1.219	0.029	Irritant	Non-Irritant
**9a**	Non-Carcinogen	11.774	0.045	0.433	0.163	Irritant	Non-Irritant
**9b**	Non-Carcinogen	3.458	0.037	0.829	0.137	Irritant	Non-Irritant
**9c**	Non-Carcinogen	2.160	0.056	0.531	0.073	Irritant	Non-Irritant
**11a**	Non-Carcinogen	22.882	0.025	1.344	0.080	Irritant	Non-Irritant
**11b**	Non-Carcinogen	6.639	0.021	2.522	0.067	Irritant	Non-Irritant
**11c**	Non-Carcinogen	4.081	0.031	1.603	0.035	Irritant	Non-Irritant
**Erlotinib**	Non-Carcinogen	8.057	0.083	0.662	0.036	Irritant	Non-Irritant

^a^Unit: mg/kg body weight/day.

^b^Unit: g/kg body weight.

#### 2.3.3. ADMET studies

**Figure 13.**
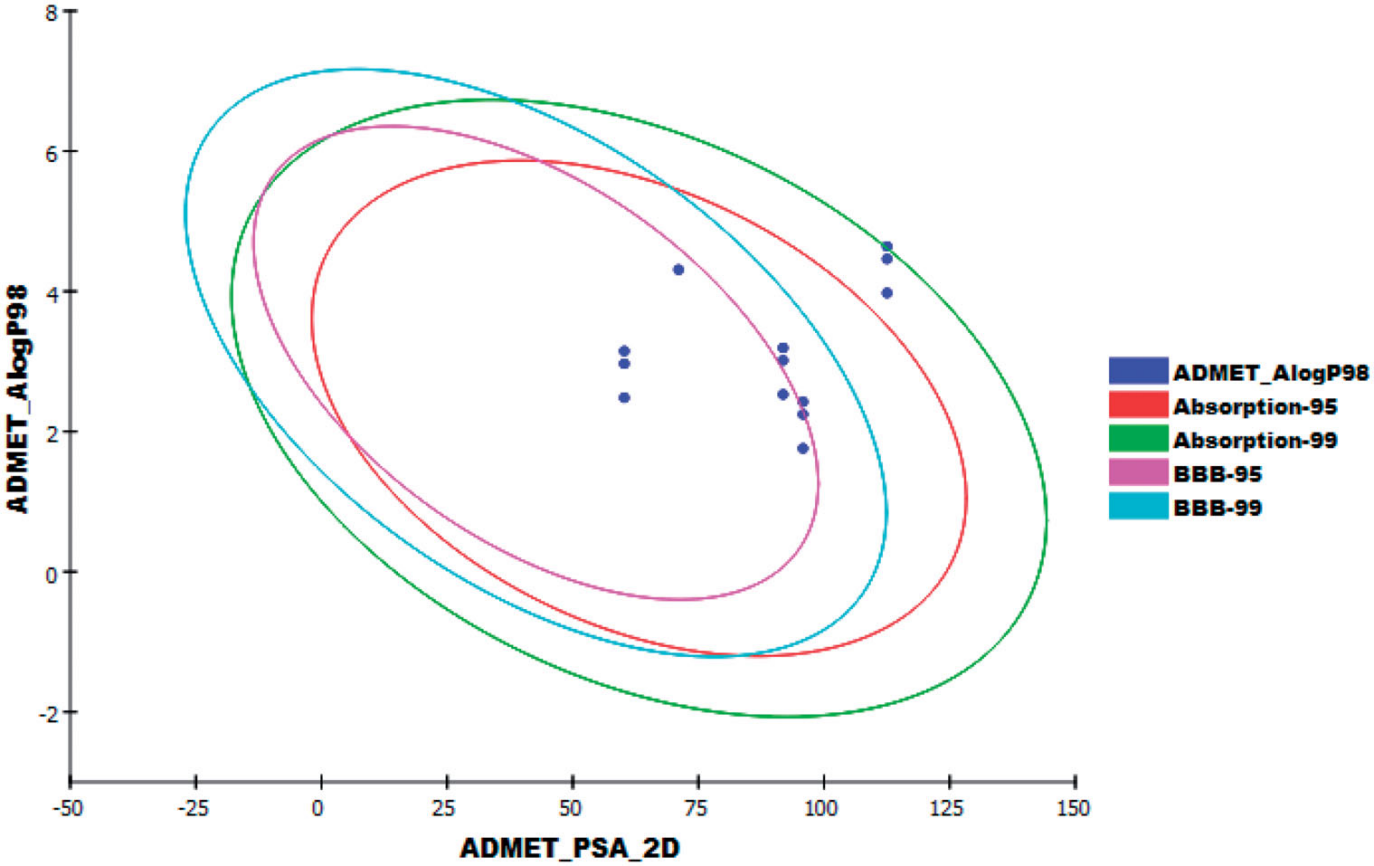
The expected ADMET study.

ADMET parameters were predicted using discovery studio software^69^^,^^73–75^. Erlotinib was used as a reference compound. The predicted ADMET parameters were listed in Table 5. The results revealed that compounds **6a–c** had very low Blood Brain Barrier penetration power. Compounds **8a–c** and **11a–c** were anticipated to have low levels of BBB penetration. On the other hand, compound **9a–c** was predicted to have medium levels of BBB penetration. Accordingly, the synthesised compounds were expected to be safe for CNS. The predicted aqueous solubility of the synthesised compounds ranged from good to low. All the synthesised compounds showed good absorption levels except compounds **6a** and **6b** which showed moderate absorption levels. All the synthesised members were predicted as non-inhibitors of CYP2D6. Additionally, all of them were expected to bind plasma protein by more than 90% (Figure 13).

**Table 5.** Predicted ADMET profile for the synthesised compounds

Comp.	BBB level^a^	Solubility level^b^	Absorption level^c^	CYP2D6 prediction^d^	PPB prediction^e^
**6a**	4	2	1	False	True
**6b**	4	2	1	False	True
**6c**	4	2	2	False	True
**8a**	3	3	0	False	True
**8b**	3	2	0	False	True
**8c**	3	2	0	False	True
**9a**	2	2	0	False	True
**9b**	2	2	0	False	True
**9c**	2	2	0	False	True
**11a**	3	3	0	False	True
**11b**	3	3	0	False	True
**11c**	3	2	0	False	True
**Erlotinib**	1	2	0	False	True

^a^BBB level, blood brain barrier level, 0 = very high, 1 = high, 2 = medium, 3 = low, 4 = very low.

^b^Solubility level, 1 = very low, 2 = low, 3 = good, 4 = optimal.

^c^Absorption level, 0 = good, 1 = moderate, 2 = poor, 3 = very poor.

^d^CYP2D6, cytochrome P2D6, TRUE = inhibitor, FALSE = non inhibitor.

^e^PBB, plasma protein binding, FALSE means less than 90%, TRUE means more than 90%.

## 3. Conclusion

In this work new compounds having the essential pharmacophoric features of EGFR inhibitors were designed and synthesised. Three series of 1*H*-pyrrole, pyrrolo[3,2-*d*]pyrimidine and pyrrolo[3,2-*e*][1, 4]diazepine derivatives were obtained. Compound **9c**, the fused pyrimido[4,5-*b*]pyrrolizine derivative, showed selectivity towards the HCT116 colon cancer cell line with activity comparable with that of DOX (IC_50_ = 0.009 and 0.008 µM, respectively). While compound **8b**, the 1*H*-pyrrole derivative featuring the extended 2-(piperidin-1-yl)acetamido moiety at C-6, showed a broad-spectrum inhibition against Hep3B, HCT116 and MCF-7cell lines (IC_50_ = 0.049, 0.031 and 0.043 µM, respectively). Regarding compound **8b**, the kinase profiling evaluations revealed its inhibitory activity against three types of kinases: CDK2/Cyclin A1, DYRK3 and GSK3 alpha. Therefore, this study reinforces the promising anticancer efficacy of 1*H*-pyrrole pyrrolo[3,2-*d*]pyrimidine derivatives based on their multi-targets activity, which were supported by molecular modelling characteristics. Add to that, their superior predicted safety and predicted pharmacokinetic properties as drug-like or lead-like molecules.

## 4. Experimental protocol

### 4.1. Chemistry

All details of chemical reagents and apparatus were described in Supplementary Data. Compounds **2**^61^**, 4a–c**^63^**, 5a–c**,^63^ **7a–c** and **10a–c**^76^ were prepared using the previously reported methods.

#### 4.1.1. General method for preparation of compounds (6a–c)

To a solution of 4-nitrobenzaldehyde (3.75 mmol) in absolute ethanol (20 ml), the pyrrolizines **5a–c** (3.75 mmol) and 0.5 ml of glacial acetic acid were added. The reaction mixture was refluxed for 4 h. The reaction mixture was left to cool. The products **6a–c** were crystallised out as orange crystals and recrystallized from ethanol

##### 4.1.2. (EZ)-7-Cyano-6-((4-nitrobenzylidene)amino)-N-phenyl)-2,3-dihydro-1H-pyrrolizine-5-carboxamide (6a)

The title compound was obtained from compound **5a** as orange crystals, m.p. 278–80 °C, yield 76%, IRʋ_max_/cm^−1^3279 (NH), 3076 (C-H aromatic), 2935, 2852 (CH_2_), 2210 (CN), 1658 (CO), 1601 (C = N), 1548, 1469 (C = C, NH), 1412, 1337 (C-N, C-O). ^1^H NMR (CDCl_3_, 400 MHz): *δ* 2.60 (m, 2H, CH_2_-2), 3.07 (t, 2H, *J* = 7.4 Hz, CH_2_-1), 4.57 (t, 2H, *J* = 7.4 Hz, CH_2_-3), 7.17 (t, 1H, *J* = 8.2 Hz, CH-4′), 7.41 (t, 2H, *J* = 8.2 Hz, CH-3′, −5′), 7.62 (d, 2H, *J* = 8.2 Hz, CH-2′, −6′), 8.1 and 8.41 (two d, 4 H, *J* = 8 Hz, CH-2′', −3′', −4′', −5′', −6′'), 9.28 (s, H, N=CH) and 10.40 (s, 1H, NH, disappeared on deuteration). ^13 ^C NMR (CDCl_3_, 100 MHz): *δ* 24.5, 25.4, 50.4, 76.7, 116.0, 119.1, 119.6, 124.4, 124.4, 129.1, 129.3, 137.8, 137.9, 140.9, 148.9, 149.7, 156.4, 158. MS (EI): m/z (%) 400 (M^+^+1, 13), 399 (M^+^, 35), 307 (100). Anal. Calcd. for C_22_H_17_N_5_O_3_ (399.40):C, 66.16; H, 4.29; N, 17.53. Found: C, 68.35; H, 4.15; N, 17.23.

##### 4.1.3. (EZ)-7-Cyano-6-((4-nitrobenzylidene)amino)-N-(4-tolyl)-2,3-dihydro-1H-pyrrolizine-5-carboxamide (6b)

The title compound was obtained from compound **5b** as orange crystals, m.p. 291–3 °C, yield 79%, IRʋ_max_/cm^−1^3280 (NH), 3069 (C-H aromatic), 2918 (CH_3_, CH_2_), 2210 (CN), 1657 (CO), 1601 (C = N), 1544, 1519 (C = C,N-H), 1411, 1338 (C-N, C-O). ^1^H NMR (CDCl_3_, 400 MHz): *δ* 2.37 (s, 3H, CH_3_Ph), 2.60 (m, 2H, CH_2_-2), 3.10 (t, 2H, *J* = 7.4 Hz, CH_2_-1), 4.59 (t, 2H, *J* = 7.4 Hz, CH_2_-3), 7.21 (d, 2H,*J* = 8.4 Hz, CH-3′, −5′), 7.53 (d, 2H,*J* = 8.4 Hz, CH-2′, −6′), 8.10 (d, 2H,*J* = 8.4 Hz, CH-2′', −6′'), 8.39 (d, 2H,*J* = 8.4 Hz, CH-3′', −5′'), 9.28 (s, H, N=CH) and 10.37 (s, 1H, NH, disappeared on deuteration). ^13 ^C NMR (CDCl_3_, 100 MHz): *δ* 20.9, 24.5, 25.5, 50.3, 76.7, 116.1, 119.3, 119.6, 124.4, 129.1, 129.8, 134.1, 135.4, 137.7, 140.9, 148.7, 149.6, 156.3, 157.9.MS (EI): m/z (%) 414 (M ^+ 1^, 15), 413 (M^+^, 51), 307 (100). Anal. Calcd. for C_23_H_19_N_5_O_3_ (413.43): C, 66.82; H, 4.63; N, 16.94. Found: C, 67.01; H, 4.58; N, 16.91.

##### 4.1.4. (EZ)-N-(4-Chlorophenyl)-7-cyano-6-((4-nitrobenzylidene)amino)-2,3-dihydro-1H-pyrrolizine-5-carboxamide (6c)

The title compound was obtained from compound **5c** as yellow crystals, m.p. 283–5 °C, yield 83%, IRʋ_max_/cm^−1^3245 (NH), 3111 (C-H aromatic), 2968 (CH_2_), 2214 (CN), 1680 (CO), 1597 (C = N), 1541, 1482 (C = C, NH), 1412, 1344 (C-N, C-O), 836, 754 (C-Cl). ^1^H NMR (CDCl_3_, 400 MHz): *δ* 2.63 (m, 2H, CH_2_-2), 3.11 (t, 2H, *J* = 7.4 Hz, CH_2_-1), 4.58 (t, 2H, *J* = 7.4 Hz, CH_2_-3), 7.38 (d, 2H,*J* = 8.4 Hz, CH-3′, −5′), 7.60 (d, 2H,*J* = 8.4 Hz, CH-2′, −6′), 8.10 (d, 2H,*J* = 8.4 Hz, CH-2′', −6′'), 8.42 (d, 2H, *J* = 8.4 Hz, CH-3′', −5′'), 9.31 (s, H, N=CH) and 10.45 (s, 1H, NH, disappeared on deuteration). ^13 ^C NMR (CDCl_3_, 100 MHz): *δ* 24.5, 25.4, 50.4, 76.7, 115.9, 119.2, 119.8, 124.4, 129.1, 129.3, 135.4, 137.6, 137.7 140.9, 148.9, 149.7, 156.4, 157.9 MS (EI): m/z (%) 435 (M ^+ 2^, 74), 433 (M^+^, 76), 405 (100). Anal. Calcd. for C_22_H_16_ClN_5_O_3_ (433.85):C, 60.91; H, 3.72; N, 16.14. Found: C, 61.10; H, 3.68; N, 16.11.

#### 4.1.5. General method for preparation of compounds (8a–c)

A mixture of compounds **7a–c** (2.91 mmol), piperidine (0.5 g, 5.9 mmol) and dry sodium bicarbonate (0.5 g, 5.9 mmol) in absolute ethanol (10 ml) was refluxed for 8 h. Then, the reaction mixture was filtered while hot, and the produced white crystals upon concentration were collected, dried, and recrystallized from ethanol.

##### 4.1.5.1. 7-Cyano-N-phenyl-6–(2-(piperidin-1-yl)acetamido)-2,3-dihydro-1H-pyrrolizine-5-carboxamide (8a)

The title compound was obtained from compound **7a** as white crystals, m.p. 205–7 °C, yield 72%, IRʋ_max_/cm^−1^3243, 3130 (NHs), 3060 (C-H aromatic), 2935, 2852 (CH_2_), 2220 (CN), 1660 (COs), 1601, 1557, 1493 (C = C, NH), 1451, 1390, 1319 (C-N, C-O). ^1^H NMR (CDCl_3_, 400 MHz):*δ* 1.5 (m, 2H, CH_2_-4′'), 1.7 (m, 4H, CH_2_-3′', −5′'), 2.54 (m, 2H, CH_2_-2), 2.69 (t, 4H,*J* = 5.2 Hz, CH_2_-2′', −6′'), 3.02 (t, 2H, *J* = 7.6 Hz, CH_2_-1), 3.27 (s, 2H, COCH_2_), 4.40 (t, 2H, *J* = 7.2 Hz, CH_2_-3), 7.12 (t, 1H, *J* = 8 Hz, CH-4′), 7.34 (t, 2H, *J* = 8 Hz, CH-3′, −5′), 7.6 (d, 2H, *J* = 8 Hz, CH-2′, CH-6′), 9.44 (s, H, NHCOCH_2_, which disappeared on deuteration) and 9.80 (s, H, CONH phenyl, which disappeared on deuteration). ^13 ^C NMR (CDCl_3_, 100 MHz): *δ* 23.5, 25, 25.7, 26.1, 49.6, 55.1, 62.1, 83.9, 114, 119.5, 120.3, 124.1, 124.4, 129, 138.3, 145.7, 157.6, 173.2. MS (EI): m/z (%) 392 (M^+^+1, 57), 391 (M^+^, 67), 138 (100).Anal. Calcd. forC_22_H_25_N_5_O_2_ (391.47): C, 67.50; H, 6.44; N, 17.89. Found: C, 67.80; H, 6.72; N, 17.67.

##### 4.1.5.2. 7-Cyano-6–(2-(piperidin-1-yl)acetamido)-N-(4-tolyl)-2,3-dihydro-1H-pyrrolizine-5-carboxamide (8b)

The title compound was obtained from compound **7b** as white crystals, m.p. 178–80 °C, yield 72%, IRʋ_max_/cm^−1^3240, 3186 (NHs), 3064 (C-H aromatic), 2983, 2930, 2853 (CH_3_, CH_2_), 2222 (CN), 1656 (COs), 1604, 1547, 1513 (C = C, NH), 1455, 1385, 1321 (C-N, C-O). ^1^H NMR (CDCl_3_, 400 MHz): *δ* 1.51 (m, 2H, CH_2_-4′'), 1.72 (m, 4H, CH_2_-3′' −5′'), 2.33 (s, 3H,CH_3_Ph), 2.54 (m, 2H, CH_2_-2), 2.70 (t, 4H,*J* = 5.2 Hz, CH_2_-2′' −6′'), 3.02 (t, 2H, *J* = 7.4 Hz, CH_2_-1), 3.28 (s, 2H, COCH_2_), 4.39 (t, 2H, *J* = 7.2 Hz, CH_2_-3),7.13 (d, 2H, *J* = 8 Hz, CH-3′, −5′), 7.49 (d, 2H, *J* = 8 Hz, CH-2′, −6′), 9.42 (s, H, NHCOCH_2_, disappeared on deuteration) and 9.69 (s, H, CONH phenyl, disappeared on deuteration). ^13 ^C NMR (CDCl_3,_ 100 MHz): *δ* 20.9, 23.5, 25.1, 25.7, 26.1, 49.5, 55.1, 62, 83.9, 114.1, 119.5, 120.4, 124.2, 129.5, 133.8, 135.8, 145.6, 157.5, 173.2. MS (EI): m/z (%) 407 (M^+^+2, 69), 406 (M^+^+1, 82), 405 (M^+^, 68) 148 (100).Anal. Calcd. for C_23_H_27_N_5_O_2_(405.49): C, 68.13; H, 6.71; N, 17.27. Found: C, 68.33; H, 6.59; N, 17.50.

##### 4.1.5.3. N-(4-Chlorophenyl)-7-cyano-6–(2-(piperidin-1-yl)acetamido)-2,3-dihydro-1H-pyrrolizine-5-carboxamide (8c)

The title compound was obtained from compound **7c** as white crystals, m.p. 210–2 °C, yield 74%, IRʋ_max_/cm^−1^3233, 3179 (NHs), 3053 (C-H aromatic), 2986, 2932 (CH_2_), 2222 (CN), 1658 (COs), 1602, 1546 (C = C, NH), 1489, 1456, 1387 (C-N, C-O), 829, 792 (C-Cl). ^1^H NMR (CDCl_3_, 400 MHz):*δ* 1.51 (m, 2H, CH_2_-4′'), 1.74 (m, 4H, CH_2_-3′' −5′'), 2.55 (m, 2H, CH_2_-2), 2.74 (t, 4H, *J* = 5.2 Hz, CH_2_-2′' −6′'), 3.02 (t, 2H, *J* = 7.4 Hz, CH_2_-1), 3.32 (s, 2H, COCH_2_), 4.39 (t, 2H, *J* = 7.4 Hz, CH_2_-3), 7.28–7.58 (4H, aromatic protons), 9.41 (s, H, NHCOCH_2_, disappeared on deuteration) and 9.93 (s, H, CONH phenyl, disappeared on deuteration). ^13 ^C NMR (CDCl_3_, 100 MHz): *δ*23.4, 25, 25.6, 26.1, 49.5, 55, 62, 83.9, 113.9, 120, 120.7, 124.4, 129, 131.6, 136.9, 145.8, 157.6, 173.3. MS (EI): m/z (%) 426 (M^+^+1, 0.9), 98 (100). Anal. Calcd. for C_22_H_24_ClN_5_O_2_ (425.91): C, 62.04; H, 5.68; N, 16.44. Found: C, 62.30; H, 5.90; N, 16.15.

#### 4.1.6. General method for preparation of compounds (9a–c)

A mixture of 6-amino-7-cyano-*N*-phenyl-2,3-dihydro-1*H*-pyrrolizine-5-carboxamide **5a–c** (3.75 mmol) and excess triethyl orthoformate was refluxed for 12 h. The solvent was then removed using a rotary evaporator and the obtained residue was crystallised from ethanol.

##### 4.1.6.1. Methyl-4-oxo-3-phenyl-4,6,7,8-tetrahydro-3H-pyrimido[4,5-b]pyrrolizine-9-carbonitrile (9a)

The title compound was obtained from compound **5a** as white crystals, m.p. 243–5 °C, yield 55%, IRʋ_max_/cm^−1^3061 (C-H aromatic), 2966, 2918 (CH_3_, CH_2_), 2214 (CN), 1693 (CO), 1524 (C = C), 1421, 1304 (C-N, C-O).^1^H NMR (CDCl_3_, 400 MHz): *δ* 2.26 (s, 3H, CH_3_C = N), 2.67 (m, 2H, CH_2_-7), 3.19 (t, 2H, *J* = 7.4 Hz, CH_2_-8), 4.40 (t, 2H, *J* = 7.2 Hz, CH_2_-6),7.25 (d, 2H, *J* = 8 Hz, CH-2′, −6′) and 7.51–7.59 (m, 3H, CH-3′, −4′, −5′). ^13 ^C NMR (CDCl_3_, 100 MHz): *δ* 24.3, 25.1, 26.2, 48, 81.1, 113.4, 113.9, 128, 129.4, 130, 137.3, 148.4, 152.3, 154.1, 154.3. MS (EI): m/z (%) 291 (M^+^+1, 3), 290 (M^+^, 15), 85 (100). Anal. Calcd. for C_17_H_14_N_4_O (290.32): C, 70.33; H, 4.86; N, 19.30. Found: C, 70.12; H, 4.90; N, 19.41.

##### 4.1.6.2. 2-Methyl-4-oxo-3–(4-tolyl)-4,6,7,8-tetrahydro-3H-pyrimido[4,5-b]pyrrolizine-9-carbonitrile (9b)

The title compound was obtained from compound **5b** as white crystals, m.p. 251–3 °C, yield 59%, IRʋ_max_/cm^−1^3073 (C-H aromatic), 2924 (CH_3_, CH_2_), 2208 (CN), 1694 (CO), 1591 (C = C), 1427, 1302 (C-N, C-O). ^1^H NMR (CDCl_3_, 400 MHz): *δ* 2.27 (s, 3H, CH_3_C = N), 2.46 (s, 3H, CH_3_Ph), 2.66 (m, 2H, CH_2_-7), 3.18 (t, 2H, *J* = 7.6 Hz, CH_2_-8), 4.41 (t, 2H, *J* = 7.2 Hz, CH_2_-6), 7.12 and 7.37 (two d, 4 H, *J* = 8 Hz, *p*-substituted phenyl ring). ^13 ^C NMR (CDCl_3_, 100 MHz): *δ*21, 24.3, 25.1, 26.2, 48, 81.1, 113.5, 114, 127.9, 130.5, 137.2, 139.4, 148.5, 152.3, 154.1, 154.3. MS (EI): m/z (%) 305 (M^+^+1, 7), 304 (M^+^, 51), 122 (100). Anal. Calcd. for C_18_H_16_N_4_O (304.35): C, 71.04; H, 5.30; N, 18.41. Found: C, 70.78; H, 5.50;N, 18.30.

##### 4.1.6.3. 3–(4-Chlorophenyl)-2-methyl-4-oxo-4,6,7,8-tetrahydro-3H-pyrimido[4,5-b]pyrrolizine-9-carbonitrile (9c)

The title compound was obtained from compound **5c** as white crystals, m.p. 268–70 °C, yield 60%, IRʋ_max_/cm^−1^3060 (C-H aromatic), 2924 (CH_3_, CH_2_), 2223 (CN), 1689 (CO), 1533 (C = C), 1419, 1300 (C-N, C-O), 840, 774 (C-Cl). ^1^H NMR (CDCl_3_, 400 MHz): *δ* 2.25 (s, 3H, CH_3_C = N), 2.67 (m, 2H, CH_2_-7), 3.19 (t, 2H, *J* = 7.2 Hz, CH_2_-8), 4.39 (t, 2H, *J* = 7 Hz, CH_2_-6), 7.19 and 7.54 (two d, 4 H, *J* = 8 Hz, *p*-substituted phenyl ring). ^13 ^C NMR (CDCl_3,_ 100 MHz): *δ* 24.4, 25.2, 26.2, 48.1, 81.3, 113.3, 113.8, 129.5, 130.3, 135.6, 135.8, 148.4, 152.6, 153.7, 154.2. MS (EI): m/z (%) 324 (M^+^, 1), 91 (100).Anal. Calcd. for C_17_H_13_ClN_4_O (324.76): C, 62.87; H, 4.03; N, 17.25. Found: C, 62.69; H, 3.97; N, 17.55.

#### 4.1.7. General method for preparation of compounds (11a–c)

To a solution of diazepino[5,6-*b*]pyrrolizine-10-carbonitrile derivative **10a–c** (3.26 mmol) in acetone, ethyl chloroacetate (0.4 g, 3.26 mmol) and anhydrous potassium carbonate (0.45 g, 3.26 mmol) was added. After refluxing for 6 h, the mixture was filtered and left to cool. The obtained white crystals were collected and recrystallized from ethanol.

##### 4.1.7.1. Ethyl-2–(10-cyano-2,5-dioxo-4-phenyl-2,3,4,5,8,9-hexahydro-[1, 4]diazepino[5,6-b]pyrrolizin-1(7H)-yl)acetate (11a)

The title compound was obtained from compound **10a** as white crystals, m.p. 179–81 °C, yield 75%, IRʋ_max_/cm^−1^3059 (C-H aromatic), 2977 (CH_2_), 2220 (CN), 1740, 1693, 1643 (COs), 1549 (C = C), 1461, 1369, 1306 (C-N, C-O). ^1^H NMR (CDCl_3_, 400 MHz): *δ* 1.29 (t, 3H, *J* = 7.2 Hz, CH_3_CH_2_),2.59 (m, 2H, CH_2_-8), 3.08 (t, 2H, *J* = 7.4 Hz, CH_2_-9), 4.24 (q, 2H, *J* = 7.2 Hz, CH_2_CH_3_), 4.39 (t, 2H, *J* = 7.2 Hz, CH_2_-7), 4.49 (s, 2H, CH_2_-3), 4.76 (s, 2H, NCH_2_CO) and 7.29–7.46 (m, 5H, aromatic protons). ^13 ^C NMR (CDCl_3_, 100 MHz): *δ* 14, 25.1, 25.4, 48.3, 49.3, 54.5, 61.9, 79.1, 114.2, 114.5, 125.7, 127.2, 129.2, 134.2, 141.6, 148.4, 159.6, 166.5, 167.5. MS (EI): m/z (%) 393 (M ^+ 1^, 1), 392 (M^+^, 1), 106 (100).Anal. Calcd. for C_21_H_20_N_4_O_4_(392.41): C, 64.28; H, 5.14; N, 14.28. Found: C, 64.58; H, 5.36; N, 14.25.

##### 4.1.7.2. Ethyl-2–(10-cyano-2,5-dioxo-4–(4-tolyl)-2,3,4,5,8,9-hexahydro-[1, 4]diazepino[5,6-b]pyrrolizin-1(7H)-yl)acetate (11b)

The title compound was obtained from compound **10b** as white crystals, m.p. 213–5 °C, yield 71%, IRʋ_max_/cm^−1^3077 (C-H aromatic), 2970, 2924 (CH_3_, CH_2_), 2220 (CN), 1741, 1690, 1642 (COs), 1548, 1465 (C = C), 1371, 1307 (C-N, C-O). ^1^H NMR (CDCl_3_, 400 MHz): *δ* 1.28 (t, 3H, *J* = 7.2 Hz, CH_3_CH_2_), 2.37 (s, 3H, CH_3_Ph),2.58 (m, 2H, CH_2_-8), 3.07 (t, 2H, *J* = 7.4 Hz, CH_2_-9), 4.24 (q, 2H, *J* = 7.2 Hz, CH_2_CH_3_), 4.40 (t, 2H, *J* = 7.2 Hz, CH_2_-7), 4.46 (s, 2H, CH_2_-3), 4.75 (s, 2H, NCH_2_CO), 7.21 and 7.30 (two d, 4 H, *J* = 8.4 Hz, *p*-substituted phenyl ring). ^13 ^C NMR (CDCl_3_, 100 MHz): *δ* 14, 21, 25.1, 25.4, 48.3, 49.3, 54.6, 61.9, 79.1, 114.2, 114.6, 125.5, 129.8, 134.2, 137.2, 139, 148.2, 159.7, 166.5, 167.6.MS (EI): m/z (%) 407 (M^+^+1, 14), 406 (M^+^, 52), 186 (100). Anal. Calcd. for C_22_H_22_N_4_O_4_(406.43): C, 65.01; H, 5.46; N, 13.78. Found: C, 65.30; H, 5.71; N, 13.77.

##### 4.1.7.3. Ethyl-2–(4-(4-Chlorophenyl)-10-cyano-2,5-dioxo-2,3,4,5,8,9-hexahydro-[1, 4]diazepino[5,6-b]pyrrolizin-1(7H)-yl)acetate (11c)

The title compound was obtained from compound **10c** as white crystals, m.p. 220–1 °C, yield 78%, IRʋ_max_/cm^−1^3092 (C-H aromatic), 2970 (CH_2_), 2222 (CN), 1749, 1686, 1647 (COs), 1549 (C = C), 1490, 1471, 1373, 1306 (C-N, C-O), 838, 772 (C-Cl). ^1^H NMR (CDCl_3_, 400 MHz): *δ* 1.28 (t, 3H, *J* = 7.2 Hz, CH_3_CH_2_), 2.60 (m, 2H, CH_2_-8), 3.09 (t, 2H, *J* = 7.6 Hz, CH_2_-9), 4.24 (q, 2H, *J* = 7.2 Hz, CH_2_CH_3_), 4.39 (t, 2H, *J* = 7.2 Hz, CH_2_-7), 4.49 (s, 2H, CH_2_-3), 4.75 (s, 2H, NCH_2_CO), 7.38 and 7.43 (two d, 4 H, *J* = 8.4 Hz, *p*-substituted phenyl ring). ^13 ^C NMR (CDCl_3_, 100 MHz): *δ* 14.1, 25.2, 25.5, 48.4, 49.4, 54.4, 62, 79.3, 114.1, 114.4, 126.9, 129.4, 132.8, 134.4, 140, 148.6, 159.6, 166.4, 167.5. MS (EI): m/z (%) 428 (M^+^+2, 23), 426 (M^+^, 64), 186 (100). Anal. Calcd. for C_21_H_19_ClN_4_O_4_(426.85): C, 59.09; H, 4.49; N, 13.13. Found: C, 59.32; H, 4.39; N, 13.10.

### 4.2. Biological evaluation

#### 4.2.1. *In vitro* anticancer screening

Cytotoxic activities evaluation of the synthesised compounds were done using the sulforhodamine B (SRB) method following the previous reported method^65^ described in Supplementary data.

#### 4.^2^.^2^. Kinase profiling assay

Compound **8b** was selected to evaluate its inhibitory activity against 20 kinases according to the reported method.^66^ The kinases inhibition assay was done by KINEXUS Corporation, Vancouver, BC, Canada, using the radiolabeled ATP determination method. Imatinib was used as a reference drug and blank control was set up and the corrected activity for the protein kinase target was determined. The results were presented as % inhibition, Table 3.

#### 4.2.3. Perturbation of cell cycle analysis

Cell cycle distribution analysis was performed for compounds **6a** and **8b** based on the previously described method in Supplementary data^77^.

### 4.3. *In silico* studies

#### 4.3.1. Docking studies

Crystallographic structures of EGFR CDK-2, and DYRK3, and GSK3 were retrieved from Protein Data Bank [PDB ID: 4HJO, resolution 2.75 Å and PDB ID: 6GUH, resolution 1.50 Å, PDB: 5Y86, resolution 1.90 Å, PDB: 5HLP, resolution 2.45 Å respectively] (http://www.pdb.org), and considered as targets for docking simulations. The docking analysis was performed using MOE software^78–80^ to evaluate the free energies and binding mode of the designed molecules against EGFR^,^ CDK-2 and DYRK3, and GSK3. At first, the crystal structures of EGFR, CDK-2 and DYRK3, and GSK3 were prepared by removing water molecules and retaining only one chain and their co-crystallised ligands, erlotinib and AZD5438, HRM, and 65 A, respectively. Then, the protein structures were protonated, and the hydrogen atoms were hidden. Next, the energy was minimised, and the binding pockets of the protein were defined.

The 2D structures of the synthesised compounds and the co-crystallised ligands, erlotinib and AZD5438, HRM, and 65 A were sketched using ChemBioDraw Ultra 14.0 and saved as MDL-SD format. Then, the saved files were opened using MOE and 3 D structures were protonated. Next, energy minimisation was applied. Before docking the synthesised compounds, validation of the docking protocol was carried out by running the simulation only using the co-crystallised ligands and low RMSD between docked and crystal conformations. The molecular docking of the synthesised compounds and the co-crystallised ligands was performed using a default protocol. MOE docking parameters include Triangle Matcher Algorithm with two rescoring functions London dG and GBVI/WSA dG were utilised to generate 10 poses of each compound. As a result, mdb output files were generated enclosing all docking results with scoring and multiple conformations of ligands. Results were finally inspected to determine the most promising compound by visualising various interactions of ligands within the binding pocket. The output from MOE was further analysed and visualised using Discovery Studio 4.0 software. The output from MOE was further analysed and visualised using Discovery Studio 4.0 software^47^^,^^48^^,^^56^^,^^60^. Mutations, missing regions and active/inactive states of the receptors were presented in Supplementary data.

#### 4.3.2. *In silico* toxicity prediction

The toxicity parameters of the synthesised compounds were calculated using Discovery studio 4.0 as described in Supplementary data^1^^,^^57^.

#### 4.3.3. *In silico* ADMET studies

ADMET descriptors (absorption, distribution, metabolism, excretion, and toxicity) of the synthesised compounds were determined using Discovery studio 4.0 as described in Supplementary data.

## Supplementary Material

Supplemental Material
