# Supplementary material for: Design, synthesis and molecular docking of new fused 1H-pyrroles, pyrrolo[3,2-d]pyrimidines and pyrrolo[3,2-e][1, 4]diazepine derivatives as potent EGFR/CDK2 inhibitors
Source: J Enzyme Inhib Med Chem. 2022 Jul 8;37(1):1884–902. doi: 10.1080/14756366.2022.2096019 (PMC9272933; doi:10.1080/14756366.2022.2096019)
Supplement: Supplemental Material [file IENZ_A_2096019_SM4506.pdf]

Design, synthesis, molecular docking and biological activity evaluations of new fused 1*H*-pyrroles, pyrrolo[3,2-d]pyrimidines and pyrrolo[3,2-*e*][1,4]diazepine derivatives

Amany Belal [1,2,*], Nagwa M. Abdel Gawad [3,*], Ahmed B. M. Mehany [4], Mohammad A.S. Abourehab [5,6], Hazem Elkady [7], Ahmed A. Al-Karmalawy [8], Ahmed S. Ismael [1,*]

[1]Medicinal Chemistry Department, Faculty of Pharmacy, Beni-Suef University, Beni-Suef, 62514, Egypt

[2]Department of Pharmaceutical Chemistry, College of Pharmacy, Taif University, P. O. Box 11099, Taif 21944, Saudi Arabia

[3] Medicinal chemistry department, Faculty of pharmacy, Cairo University, Kasr El-Eini Street 11562, Egypt.

[4] Department of Zoology, Faculty of Science, Al-Azhar University, Nasr City, Cairo, Egypt.

[5]Department of Pharmaceutics, Faculty of Pharmacy, Umm Al-Qura University, Makkah 21955, Saudi Arabia.

[6]Department of Pharmaceutics and Industrial Pharmacy, College of Pharmacy, Minia University, Minia 61519, Egypt.

[7] Pharmaceutical Medicinal Chemistry & Drug Design Department, Faculty of Pharmacy (Boys), Al-Azhar University, Cairo11884, Egypt.

[8] Department of Pharmaceutical Medicinal Chemistry, Faculty of Pharmacy, Horus University-Egypt, New Damietta 34518, Egypt.

*** Corresponding authors:**

Dr.Amany Belal: E-mail: abilalmoh1@yahoo.com & amany.mehani@pharm.bsu.edu.eg , Dr. Nagwa M. Abdel Gawad (nagwa_gawad2010@yahoo.com) and Dr. Ahmed S. Ismael (ahmed_safwat1987@yahoo.com).

Content

**1**	The used chemicals, reagents, and different apparatus
**2**	Biological tests
**3**	*In silico* studies procedures

The used chemicals, reagents, and different apparatus

Chemical reagents were obtained from commercial sources. The solvents obtained from sigma-Aldrich were dried by standard methods. Melting points (m.p.) of the new compounds are uncorrected and were determined on IA 9100MK-Digital Melting Point apparatus using open capillary tube method. Elemental analysis (C, H and N) was determined in the micro analytical Center, Faculty of Science, Cairo University. Infrared spectra were made on BRUKER Vector 22 (Japan), infrared spectrophotometers, the IR absorption were expressed in wavenumber (cm^{-1}) using potassium bromide disc. BRUKER APX400 spectrometer at 400 MHz was used to determine the proton magnetic resonance ^{1}H NMR spectra, the samples were dissolved in the specified solvent, chemical shifts were reported on the δ scale and were related to that of the solvent and *J* values are given in Hz. ^{13}C NMR spectra were recorded on a Bruker APX400 at 100 MHz at the faculty of pharmacy, Beni-Suef University. Mass spectra were recorded on Fennigan MAT, SSQ 7000, Mass spectrometer, at 70 eV (EI), Micro analytical Center, Faculty of Science, Cairo University.

Biological testing

1. *In vitro* anti-proliferative activity (sulforhodamine B (SRB) method)

Three cancer cell lines were used in the assay: liver (Hep3B), colon (HCT116), and breast (MCF-7) cancer cell lines. They were obtained from American Type Culture Collection (ATCC, Minnesota, USA) through the Tissue Culture Unit, The Egyptian Organization for Biological Products and Vaccines (Vacsera, Egypt). The SRB assay was performed in Center of Genetic Engineering, Al-Azhar University, Cairo, Egypt. Chemical and reagents were purchased from Sigma-Aldrich.

Regarding cell preparation, the cancer cell suspensions were plated into 96-multiwell plate at 20,000 cells per well and the plate was incubated in a humidified incubator (37ºC, 95% air and 5% of CO_2) for 24 h before treatment. The test compounds (**12**, **14**, **15** and **17**) and Doxorubicin (DOX) as a reference drug with different concentrations (0, 6.25, 12.5, 25, 50 and 100 µg/mL DMSO) were added to the cells. The test was performed Triplicate for each concentration. Negative control was included. Following 48 h treatment, cells were fixed by layering 150 µL/well of 10% trichloroacetic acid (TCA) directly on the top of the incubation medium, and then the plate was incubated at 4ºC for 1 h. the wells were rinsed five times with tap water and then allowed to dry. The cells were stained with 70 µL/well of SRB (0.4% in 1% acetic acid) for 30 min and rinsed four times in 1% acetic acid to remove the unbound dye, and the plates were dried for 24 h. After drying, 150 µL/well of 10 mM Tris buffer [tris(hydroxymethyl)aminomethane, (pH 7.4)] was added and the plate was shaken for 5 min. The absorbance was measured spectrophotometrically. The IC_{50} values of compounds (**12, 14, 15 and 17**) and DOX were determined using sigmoidal concentration response curve fitting models (SigmaPlot software). Cytotoxic activities of the test compounds and DOX against liver cancer (Hep3B), colon cancer (HCT116), and breast cancer (MCF-7) cell lines were expressed as IC_{50} (µM), **Table 1**

2. Flow cytometry analysis for cell cycle

Cell cycle distribution analysis was performed for compounds **12a** and **14b** based on previously described method as follows. Propidium iodide (PI) fluorescence-labelled cell nuclei were suspended in stream of fluid, and an argon laser $A^{488}/^{645}$ was used to excite PI (emission A^{617}), and emission above A^{550} was collected. MCF-7 cells were seeded in 6 well plates at $1{\times}10^5$ cells/well in 2 mL medium and were left to attach overnight before treatment with control or drug 2 (n = 2) to final concentrations: 0, 1, 5 and 10 µM. Then plates were incubated for 72 h. After incubation, medium was collected and kept on ice. Cells were washed with ice-cold PBS (2x). Trypsin (0.5 mL) was added to each well and incubated at 37 °C for 5 min, and the detached cells were pooled with the floating cell suspension. Then tubes were centrifuged at 1200 rpm for 5 min at 4 °C and supernatant was discarded. Pellets were washed in 1 mL of PBS, centrifuged and fixed overnight in 70% ice-cold ethanol. Then pellets were centrifuged, re-suspended in PBS with addition of ribonuclease-A (15 min), followed by PI (2µL/mL). Samples were held on ice, and analysed by flow cytometry (BC, FC500). Data analysis of DNA contents (PI bound to DNA) of 20000 events was carried out using Expo 32 software. Doublets were differentiated from single cells in the G_2/M phase by gating them out manually. The results were presented in **Fig. 3** and **4**.

***In silico* studies procedures**

1. Docking studies

Crystallographic structures of EGFR and CDK-2 were retrieved from Protein Data Bank [PDB ID: 4HJO, resolution 2.75 Å and PDB ID: 6GUH, resolution 1.50 Å, respectively] (http://www.pdb.org),and considered as targets for docking simulations. The docking analysis was performed using MOE [61] software to evaluate the free energies and binding mode of the designed molecules against EGFR and CDK-2. At first, the crystal structures of EGFR and CDK-2 were prepared by removing water molecules and retaining only one chain and their co-crystallized ligands, erlotinib and AZD5438, respectively. Then, the protein structures were protonated, and the hydrogen atoms were hided. Next, the energy was minimized, and the binding pockets of the protein were defined.

The 2D structures of the synthesized compounds and the co-crystallized ligands, erlotinib and AZD5438 were sketched using ChemBioDraw Ultra 14.0 and saved as MDL-SD format.

Then, the saved files were opened using MOE and 3D structures were protonated. Next, energy minimization was applied. Before docking the synthesized compounds, validation of the docking protocol was carried out by running the simulation only using the co-crystallized ligands and low RMSD between docked and crystal conformations. The molecular docking of the synthesized compounds and the co-crystallized ligands was performed using a default protocol. In each case, 30 docked structures were generated using genetic algorithm searches. The output from of MOE was further analyzed and visualized using Discovery Studio 4.0 software.

2. *In Silico* toxicity prediction

The toxicity parameters of the synthesized compounds were calculated using Discovery studio 4.0. Erlotinib was used as a reference drug. At first, the CHARMM force field was applied then the compounds were prepared and minimized according to the preparation of small molecule protocol. Then different parameters were calculated from toxicity prediction (extensible) protocol.

3. In silico ADMET studies

ADMET descriptors (absorption, distribution, metabolism, excretion, and toxicity) of the synthesized compounds were determined using Discovery studio 4.0. At first, the CHARMM force field was applied then the compounds were prepared and minimized according to the preparation of small molecule protocol. Then ADMET descriptors protocol was applied to carry out these studies.

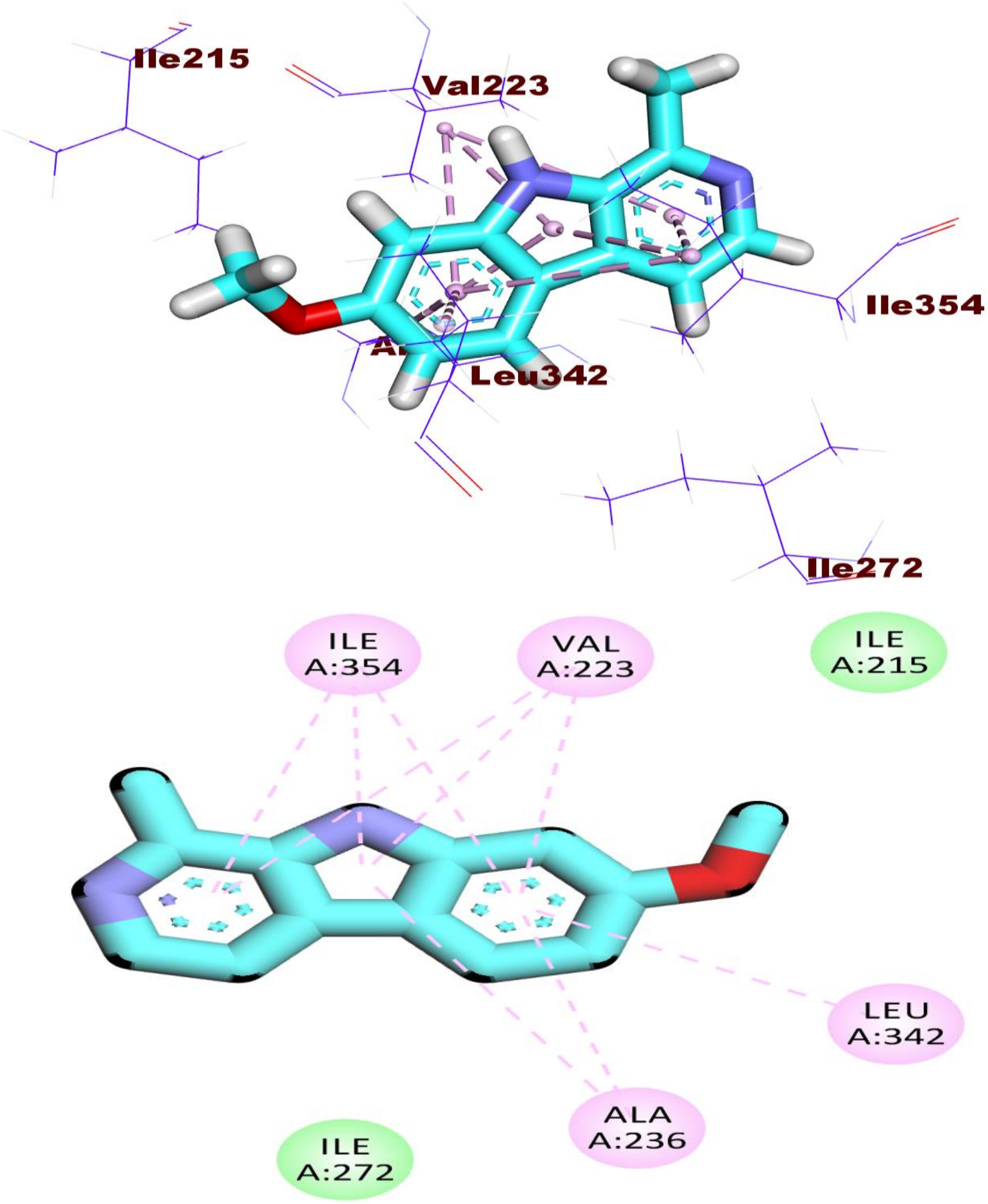

Co-crystallized ligand (HRM) docked into the active site of DYRK3.

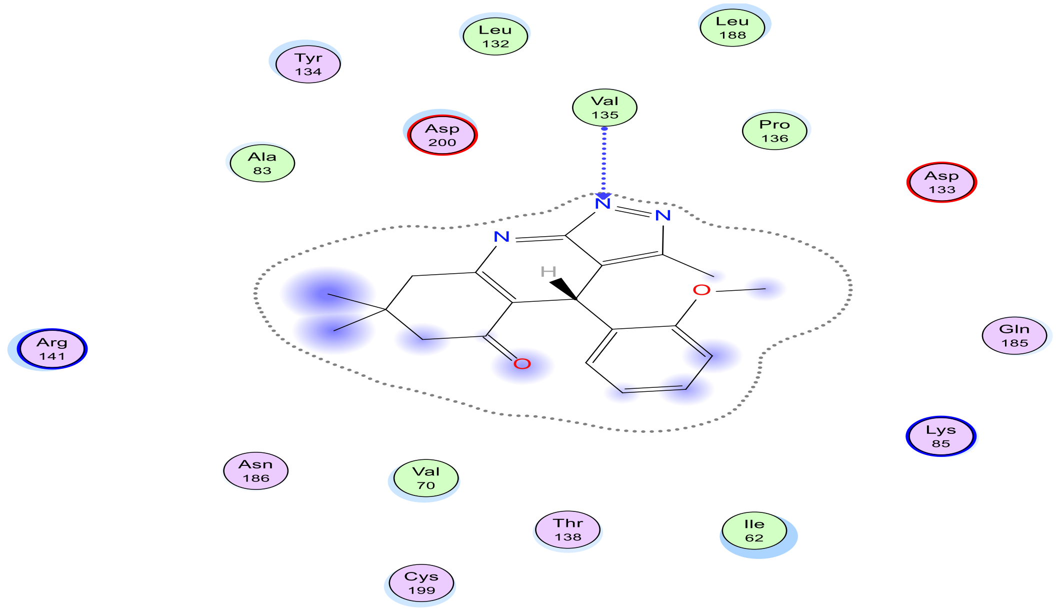

Co-crystallized ligand (65A) docked into the active site of GSK3.
